# Erythropoietin‐Stimulated Macrophage‐Derived Extracellular Vesicles in Chitosan Hydrogel Rescue BMSCs Fate by Targeting EGFR to Alleviate Inflammatory Bone Loss in Periodontitis

**DOI:** 10.1002/advs.202500554

**Published:** 2025-04-28

**Authors:** Shuchen Liu, Zhuoran Wang, Yuhuan Li, Ziyi Pan, Lei Huang, Jing Cui, Xue Zhang, Mingxi Yang, Yuan Zhang, Daowei Li, Hongchen Sun

**Affiliations:** ^1^ Jilin Provincial Key Laboratory of Tooth Development and Bone Remodeling School and Hospital of Stomatology, Jilin University 1500 Qinghua Road Changchun 130021 China; ^2^ Department of Anesthesiology The First Hospital of Jilin University 71 Xinmin Street Changchun 130021 China; ^3^ Department of Surgery Experimental Surgery CCM CVK Charité – Berlin University Medicine Free University of Berlin and Humboldt University of Berlin 10117 Berlin Germany; ^4^ Department of Orthopedics China‐Japan Union Hospital of Jilin University Changchun 130012 China; ^5^ State Key Laboratory of Supramolecular Structure and Materials College of Chemistry Jilin University Changchun 130012 China

**Keywords:** epidermal growth factor receptor (EGFR), erythropoietin, extracellular vesicles (EVs), microRNAs, periodontitis

## Abstract

Loss of periodontal tissue due to persistent inflammation in periodontitis is a major cause of tooth loss in adults. Overcoming osteogenic inhibition in the inflammatory periodontal environment and restoring the regenerative capacity of endogenous bone marrow mesenchymal stem cells (BMSCs) remain critical challenges in current treatment approaches. Macrophage‐derived extracellular vesicles (EVs) are key regulators of osteogenesis in recipient cells, yet the role of erythropoietin (EPO) in modifying macrophages and the function of their EVs in bone regeneration remain unclear. In this study, EVs from EPO‐stimulated macrophages (EPO‐EVs) are isolated, and they are encapsulated in a chitosan/β‐sodium glycerophosphate/gelatin (CS/β‐GP/gelatin) hydrogel to create a controlled‐release EVs delivery system for localized periodontal environment. EPO‐EVs restore the osteogenic function of mouse BMSCs (mBMSCs) and mitigate inflammatory bone loss in a periodontitis mouse model. Mechanistically, miR‐5107‐5p, significantly enriched in EPO‐EVs, is delivered to mBMSCs, where it suppresses epidermal growth factor receptor (EGFR) expression and alleviates EGFR's inhibitory effect on RhoA. This process counteracts osteogenic inhibition in inflammatory settings through the EGFR/RhoA axis. Overall, EVs from EPO pretreated macrophages restore the osteogenic capacity of mBMSCs under inflammation by inhibiting EGFR expression, providing new insight into therapeutic mechanisms and offering a promising approach for future periodontitis treatment.

## Graphical abstract

A therapeutic strategy is proposed for periodontitis using extracellular vesicles derived from EPO‐stimulated macrophages (EPO‐EVs). This schema demonstrates how EPO‐EVs enhance the osteogenic activity of mBMSCs in an inflammatory environment by inhibiting EGFR. The abundant miR‐5107‐5p in EVs, following EPO stimulation, alleviates inflammatory bone loss in periodontitis mouse models through the EGFR/RhoA axis.

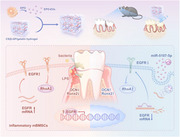

## 1 Introduction

Periodontitis is a chronic and multifactorial inflammatory disease that causes damage to the periodontium, leading to the destruction of tooth‐supporting tissues such as the alveolar bone, cementum, gingiva, and periodontal ligament.^[^ ^1^ ^]^ Approximately 45–50% of the global population is affected by periodontitis, which significantly increases the incidence of various systemic diseases, including cardiovascular diseases, diabetes, rheumatoid arthritis, and Alzheimer's disease.^[^ ^2^, ^3^ ^]^ Current treatment strategies for periodontitis primarily focus on mechanical debridement techniques, such as scaling and root planing (SRP), often supplemented with adjunctive local antimicrobials therapies to control inflammation. For surgical interventions, guided tissue regeneration (GTR) and guided bone regeneration (GBR) are commonly employed to repair damaged tissues and partially restore periodontal attachment.^[^ ^1^, ^4^, ^5^ ^]^ However, these approaches often overlook the necessity of restoring the regenerative capacity of periodontal tissues damaged by inflammation. The limited outcomes in alveolar bone formation ultimately result in tooth loss, emphasizing the urgent need to develop an effective bone regeneration treatment strategy for periodontitis.

Bone regeneration is largely dependent on the osteogenic capacity of endogenous bone marrow mesenchymal stem cells (BMSCs) due to their critical involvement in bone physiology and pathology.^[^ ^6^ ^]^ Recent advances in tissue engineering have highlighted the potential of utilizing endogenous BMSCs as a promising alternative for periodontal tissue regeneration.^[^ ^7^, ^8^, ^9^ ^]^ Compared to exogenous BMSC‐based therapies, leveraging endogenous BMSCs offers advantages such as more cost‐effective, less invasive, and a lower risk of immunogenicity.^[^ ^10^, ^11^ ^]^ However, the osteogenic function of BMSCs is frequently impaired by inflammatory stimuli within pathological microenvironments. Previous research has revealed that bacterial components in the inflammatory microenvironment can activate the epidermal growth factor receptor (EGFR) on BMSCs.^[^ ^12^ ^]^ EGFR, a key regulator in the ErbB signaling pathway, negatively influences osteoblastic differentiation,^[^ ^13^, ^14^ ^]^ identifying it as a potential therapeutic target for periodontitis. The application of chemical inhibitors targeting the tyrosine kinase domain of EGFR, such as Gefitinib, has been shown to effectively reduce inflammatory bone loss in a ligature‐induced experimental periodontitis mouse model.^[^ ^15^, ^16^ ^]^ Despite this, traditional EGFR inhibitors face significant limitations, including toxicity and the potential for drug resistance, which hinder their broader clinical application. Therefore, it is essential to establish innovative strategies to counteract the inhibitory effects of EGFR on osteogenesis, restore the function of inflamed BMSCs, enhance the endogenous regenerative niche, and promote in situ alveolar bone regeneration.

Current researches emphasize the importance of macrophage‐BMSC interactions in modulating inflammation and promoting periodontal tissue regeneration.^[^ ^17^ ^]^ Increasing evidence suggests that macrophage‐derived EVs may serve as a promising functional tool for biomaterial‐mediated endogenous bone regeneration.^[^ ^18^, ^19^ ^]^ As natural nanoscale vesicles, EVs have garnered significant attention in cell‐free therapies for periodontal tissue regeneration.^[^ ^20^, ^21^, ^22^ ^]^ Furthermore, emerging evidence highlights miRNAs as critical regulators of bone repair and regeneration. In the osteoimmune microenvironment, macrophage‐derived EVs can stably transfer miRNAs to BMSCs, modulating their proliferation, migration, differentiation, and apoptosis by modulating target gene expression at the post‐transcriptional level.^[^ ^23^ ^]^ E.g., miR‐5106, carried in EVs from M2 macrophages, promotes BMSC osteogenic differentiation by targeting SIK2 and SIK3.^[^ ^24^ ^]^ Conversely, miR‐30‐5p in EVs derived from M1 macrophages inhibits osteogenesis by downregulating Runx2, contributing to bone loss in periodontitis.^[^ ^25^ ^]^ Moreover, certain drugs or nanoparticles, such as tantalum particles and tannic acid, can enhance the expression of specific miRNAs in macrophages, promoting bone repair and wound healing.^[^ ^26^, ^27^ ^]^ These evidences demonstrated the therapeutic potential of macrophages EV‐derived miRNAs in reducing inflammation and facilitating bone regeneration. Despite these advances, the therapeutic potential of novel EVs derived from modified macrophages, as well as the alterations in their miRNAs content in BMSC‐based treatments for periodontitis, remains underexplored. Further research is needed to optimize these strategies and fully harness their potential for clinical applications.

Erythropoietin (EPO), traditionally recognized for its role in stimulating red blood cell production, has recently shown potential in promoting bone regeneration in models of alveolar defects^[^ ^28^ ^]^ and periodontitis when delivered with appropriate scaffolds.^[^ ^29^, ^30^ ^]^ Beyond its hematopoietic and osteogenic functions, EPO interacts with various immune cells by binding to the tissue protection receptor (TPR), regulating their activation and differentiation while exerting pro‐angiogenic, tissue regenerative, anti‐inflammatory, and anti‐apoptotic effects.^[^ ^31^, ^32^, ^33^ ^]^ Moreover, previous studies have demonstrated that EPO treatment leads to changes in miRNAs production in MSCs, with 70.28% of the affected miRNAs being upregulated. This upregulation contributes to the enhanced protective effects of MSCs observed in models of kidney injury.^[^ ^34^ ^]^ While the impact of EPO on miRNAs modulation within MSCs is predominantly upregulated, its effects on highly plastic immune cells—such as macrophages—remain largely unexplored. To date, the therapeutic potential of EVs derived from EPO‐stimulated macrophages, particularly in miRNA‐based therapies, is a promising yet under‐researched area. Further investigations are crucial to elucidate the influence of EPO on macrophages EV‐derived miRNAs and their potential applications in inflammation resolution, tissue regeneration, and bone repair.

One of the key limitations of EV‐based therapies is their susceptibility to degradation and instability in periodontal pockets, limiting their therapeutic efficacy. To address this, hydrogels, with their favorable biocompatibility and porous structure, have emerged as promising carriers for EVs delivery.^[^ ^35^, ^36^ ^]^ Among them, chitosan (CS) hydrogels combined with β‐sodium glycerophosphate (β‐GP) have gained significant attention in the treatment of dentistry‐related diseases due to their injectability and thermosensitive gelation at body temperature.^[^ ^37^, ^38^ ^]^ Meanwhile, the negatively charged bilayer phospholipid membrane structure of EVs facilitates their incorporation into chitosan‐based porous gels, enabling sustained slow release of EVs and extending their retention time in vivo. Additionally, the inclusion of gelatin in these hydrogels can effectively shorten the gelation time by accelerating the crosslinking between CS and β‐GP,^[^ ^39^ ^]^ enhancing their ability to fill irregular periodontal pockets more effectively. Inspired by this, incorporating specific EVs into a CS/β‐GP/gelatin hydrogel offers a more effective approach for clinical periodontitis therapy.

**Scheme 1:**
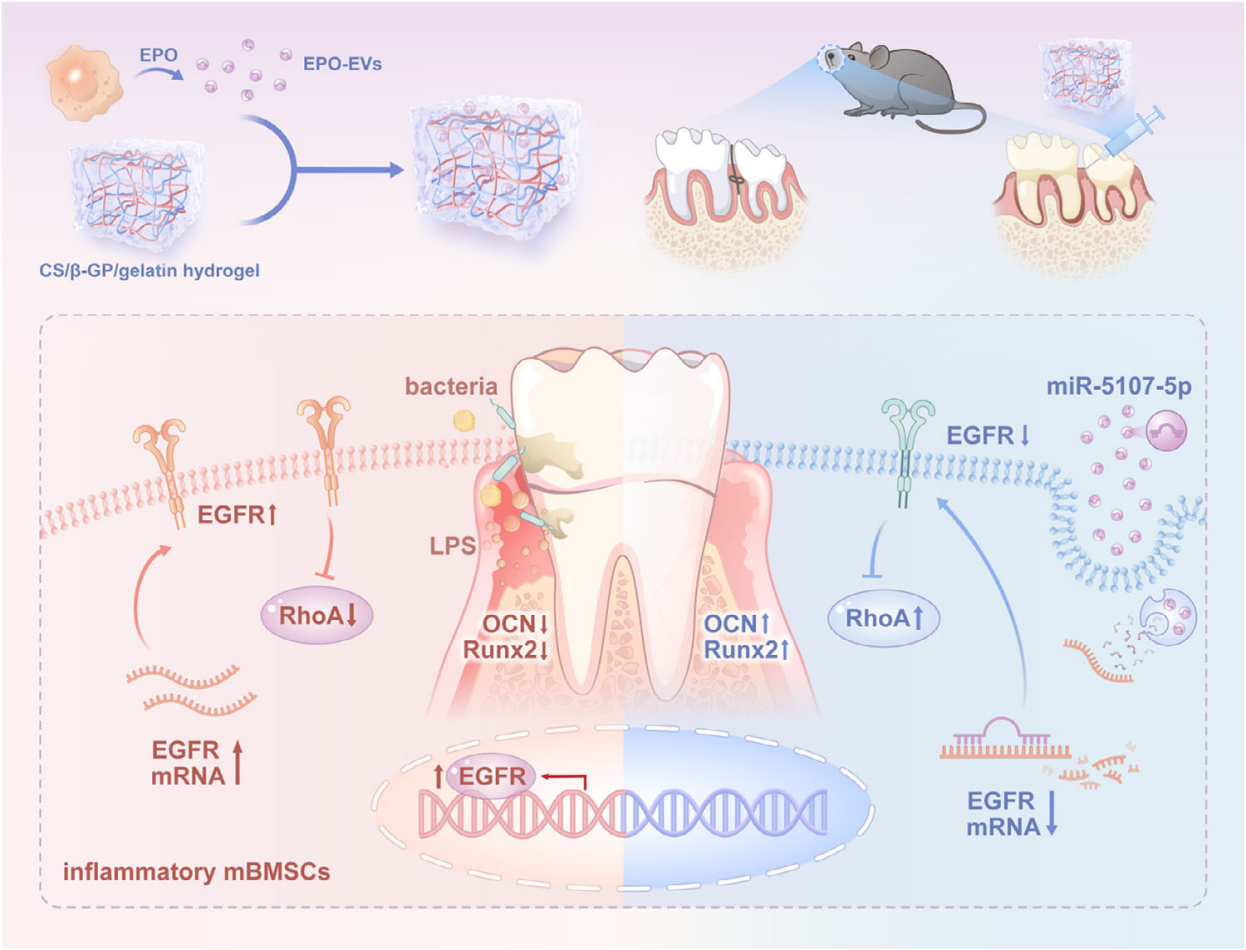
Proposed therapeutic strategy for periodontitis using EVs derived from EPO‐stimulated macrophages. The schema shows that EPO‐EVs increase the osteogenic activity of mBMSCs in the inflammatory milieu through inhibiting EGFR. The abundant EV‐derived miR‐5107‐5p after EPO stimulation alleviates the inflammatory bone loss via the EGFR/RhoA axis.

In this study, we first isolated and characterized EVs derived from EPO‐stimulated macrophages (EPO‐EVs) and subsequently incorporated them into a temperature‐sensitive CS/β‐GP/gelatin hydrogel to create the EPO‐EVs/hydrogel system. We then evaluated the effects of this system on the osteogenic differentiation of mouse bone marrow mesenchymal stem cells (mBMSCs) in vitro, as well as its ability to promote alveolar bone regeneration in a periodontitis mouse model. Sequencing analysis revealed that EPO‐EVs are highly enriched with miR‐5107‐5p, which target EGFR in mBMSCs, mitigating its inhibitory effects on osteogenesis by modulating the EGFR/RhoA signaling pathway, ultimately reducing inflammatory bone loss both in vitro and in vivo (**Scheme** **1**).

## 2 Experimental Section

### 2.1 Materials, Animals, and Cells

#### 2.1.1 Materials

Chitosan (high molecular weight, Sigma catalog number: 419419), type A gelatin (Sigma catalog number: G2625), β‐sodium glycerophosphate (β‐GP, Sigma catalog number: G9422), erythropoietin (EPO, Amresco), serum‐free medium (Umibio, UR51102), fluorescent labeling dye DiR, PKH26 and PKH67 (Umibio, UR21017, UR52302, UR52303), Calcein‐AM/PI double stain kit (Beyotime, C2015S), Cell Counting Kit‐8 (CCK‐8, Yeasen, 40203ES60), Annexin V‐FITC apoptosis detection kit (Beyotime, C1062L), BCIP/NBT staining kit (Beyotime, C3206), Alkaline Phosphatase assay kit (Beyotime, P0321S), Alizarin Red S (Oricell, ALIR‐10001), Strand cDNA Synthesis SuperMix kit (Yeasen, 11123ES60), qPCR SYBR Green Master Mix (Yeasen, 11202ES08), miRNA isolation kit, miRNA 1^st^ Strand cDNA kit, and SYBR Green qPCR kit (TIANGEN, DP501, KR211, FP411), Lipofectamine 3000 (Thermo Fisher, L3000008), dual luciferase reporter gene assay kit (GenePharma), AntagomiR‐NC, Antagomir‐5107, mimic‐5107 and inhibitor‐5107 (RiboBio, Guangzhou, China). The details about antibodies were listed in Table S1 (Supporting Information). Primers and the miRNA‐specific primers were synthesized by Shanghai Sangon, with sequences listed in Tables S2 and S3 (Supporting Information).

#### 2.1.2 Animals

6‐weeks male C57BL/6J mice were purchased from Liaoning Changsheng Biotechnology Co. Ltd. for the experiments, with a standard diet and average weight of 16 ± 1 g. All animal experiments were performed in compliance with the Guidelines for the Care and Use of Laboratory Animals of Jilin University and were approved by the Animal Ethics Committee of Jilin University (approval number: 20210642).

#### 2.1.3 Cell Culture

The RAW 264.7 and HEK‐293 cell lines were obtained from the American Type Culture Collection (ATCC) and cultured in Dulbecco's modified Eagle medium (DMEM, Gibco) with 10% fetal bovine serum (FBS, Gibco) and 1% penicillin/streptomycin (100 IU/mL, HyClone). mBMSCs were obtained from the femurs and tibias of 6 weeks C57BL/6J mice following previously described methods^[^ ^40^ ^]^ and cultured in alpha minimal essential medium (α‐MEM, Gibco) supplemented with 10% FBS and 1% penicillin/streptomycin. All three cell types were cultured at 37 °C in a humidified atmosphere containing 5% CO₂.

### 2.2 EVs Isolation

RAW 264.7 cells were cultured with 20 U/mL EPO for 24 h. Afterward, the cells were washed three times with phosphate‐buffered saline (PBS) and incubated with fresh serum‐free medium for an additional 24 h before the cell culture medium was collected. EVs isolation from the culture medium was performed according to the previously established guidelines.^[^ ^41^ ^]^ Briefly, the entire centrifugation process was maintained at 4 °C. The collected culture medium was centrifuged 10 min at 300 g to remove suspended cells, followed by 10 min at 2 000 g to remove dead cells and 30 min at 10 000 g to remove cell debris. Subsequently, the suspension was centrifuged 70 min at 100 000 g to pellet the EVs. The pelleted EVs were washed with sterile PBS and filtered through a 0.22 µm filter. Finally, the suspension was centrifuged at 100 000 g for 70 min to get purified normal control macrophages‐derived extracellular vesicles (NC‐EVs) or EPO‐EVs. The pelleted EVs were reconstituted in sterile PBS, or lysed in RIPA buffer for subsequent experiments.

### 2.3 EVs Qualification and Characterization

The isolated EVs were fixed in 2.5% glutaraldehyde at 4 °C overnight. After being washed twice with PBS, the vesicles were loaded onto copper grids coated with a carbon film and negatively stained with aqueous phosphotungstic acid. The samples were then observed using a transmission electron microscopy (TEM) operated at 80 kV. Nanoparticle tracking analysis (NTA) was used to measure the size and concentration of isolated EVs. Briefly, EV samples diluted in PBS were analyzed for 60 s using Nanosight's automatic settings, with three measurements taken. The characteristic EV surface markers, HSP70 and CD63 were identified through Western blot.

### 2.4 EVs Labeling and In Vitro Cellular Uptake of NC‐EVs and EPO‐EVs

The EVs were labeled with PKH67 Green Fluorescent Dye following the manufacturer's protocol. Briefly, the EV suspension was mixed with the PKH67 dye solution in a recommended ratio and incubated for 10 min. The PKH67‐labeled EVs were subsequently incubated with mBMSCs for 0, 3, 6, 12, or 24 h at 37 °C. Images were captured using a confocal laser scanning microscope (CLSM).

### 2.5 Synthesis of the EV‐Encapsulated CS/β‐GP/Gelatin Hydrogel

The preparation of the CS/β‐GP/gelatin hydrogel was based on previous studies.^[^ ^30^ ^]^ A 500 µg mL⁻^1^ EV solution was mixed with 56% β‐GP in a 1:1 volume ratio, resulting in a final β‐GP concentration of 28%. Subsequently, the 2% (w/v) chitosan solution, 0.5% (w/v) gelatin solution, and the β‐GP/EVs solution were mixed and incubated for 5 min at 37 °C to promote cross‐linking and form the hydrogels.

### 2.6 Characterization of NC‐EVs/Hydrogel and EPO‐EVs/Hydrogel

To examine the microstructure of the CS/β‐GP/gelatin hydrogel and the distribution of EVs within it, NC‐EVs/hydrogel and EPO‐EVs/hydrogel samples were lyophilized and then coated with gold using a sputter‐coater. The morphologies of the hydrogels were examined using a scanning electron microscope (SEM) at a primary electron energy of 3 kV. The structure and composition of the organic functional groups in the hydrogels were analyzed using Fourier transform infrared spectroscopy. The dispersion of PKH26‐labeled EVs within the hydrogels was examined using CLSM.

### 2.7 Biocompatibility Analysis of the EV‐Encapsulated CS/β‐GP/Gelatin Hydrogel

mBMSCs were cultured in media containing various hydrogel extracts (hydrogel, NC‐EVs/hydrogel, EPO‐EVs/hydrogel). At specific time points, cells were assessed using the Annexin V‐FITC apoptosis detection kit and analyzed by flow cytometry with FlowJo software. Cell viability was evaluated with a calcein‐AM/PI double stain kit according to the manufacturer's instructions, while cell proliferation was quantified with the CCK‐8 assay.

### 2.8 EVs Releasing Assay

The release profiles of NC‐EVs/hydrogel and EPO‐EVs/hydrogel were analyzed following established protocols.^[^ ^42^ ^]^ Briefly, 100 µL of NC‐EVs/hydrogel or EPO‐EVs/hydrogel was placed in the upper chamber of a 24‐well transwell, with 500 µL PBS added to the lower chamber. The setup was incubated on a shaker at 37 °C. At the specified time points, 10 µL of PBS was collected to determine the releasing EV concentration using the BCA protein assay kit, and an equiv. amount of fresh PBS was replenished in the lower chamber.

### 2.9 Osteogenic Differentiation Assay

To induce osteogenic differentiation of mBMSCs, the cells were cultured in osteogenic medium containing 10 mm β‐sodium glycerophosphate and 50 µm L‐ascorbic acid. The mBMSCs were seeded in 12‐well plates, and the osteogenic medium with hydrogel, NC‐EVs/hydrogel or EPO‐EVs/hydrogel extracts was refreshed every 3 days. On day 7 and 14, cells were fixed and ALP staining was performed using a BCIP/NBT staining kit. ALP quantification was conducted using an Alkaline Phosphatase assay kit according to the manufacturer's protocol. Calcium nodes were assessed using Alizarin Red S (ARS) staining on day 21. The fixed cells were washed three times with ddH_2_O. Alizarin Red S was then added to each well and incubated for 5 min. The plate was subsequently washed with ddH_2_O to remove excess dye and photographed. The calcified nodules in ARS‐stained samples were eluted with cetylpyridinium chloride and quantified by measuring OD values at 562 nm wavelength. Immunofluorescent staining was performed to detect Runx2, OCN, EGFR or RhoA.

### 2.10 qPCR

Total mRNA was extracted from mBMSCs using TRIzol reagent according to the manufacturer's instructions. Reverse transcription was carried out with the Strand cDNA Synthesis SuperMix kit, and qPCR was performed using the qPCR SYBR Green Master Mix. Gene expression levels were analyzed using an Agilent MX3000P system. miRNA expression was analyzed using a miRNA isolation kit, following the manufacturer's protocol to extract total EV‐derived miRNAs. To measure miRNA expression levels in EVs, cDNA synthesis was performed using the miRNA 1^st^ Strand cDNA kit, followed by qPCR analysis conducted on the CFX96 System. Mouse glyceraldehyde‐3‐phosphate dehydrogenase (GAPDH) or U6 served as the endogenous control to normalize total RNA differences among samples. Relative mRNA expression was analyzed using the 2^−∆∆CT^ method.

### 2.11 Western Blot

Protein concentrations of cells or EVs were measured using the BCA protein assay. The protein samples were denatured through boiling with 5 × loading buffer and then separated on a 10–15% SDS‐PAGE gel. Subsequently, the proteins were transferred to PVDF membranes, blocked with dried skimmed milk, and incubated with primary antibodies at 4 °C overnight. The following day, membranes were washed three times with TBST buffer and incubated for 2 h at room temperature with secondary antibodies. After washing three more times, the blot signal was detected using an enhanced chemiluminescence reagent, and band density was analyzed using ImageJ software.

### 2.12 In Vivo Animal Experiments

The mice periodontitis model was constructed based on previous studies.^[^ ^43^ ^]^ Briefly, the mice were fully anaesthetized with isoflurane to avoid discomfort. A 5‐0 silk ligature was placed between the first and second molars of the mouse for 7 days to induce periodontitis. A separate group of mice without ligatures served as the negative control. Following ligature removal, the PD mice were randomly divided into various treatment groups (*n* = 5 per group). For EV treatment, 100 µL hydrogel, NC‐EVs/hydrogel, EPO‐EVs/hydrogel, or equiv. volumes of PBS were injected locally once a week for 3 weeks. For Antagomir treatment, 10 nmol of Antagomir‐5107 or Antagomir‐NC per mouse was administered locally every 3 days. The injection dosage was determined based on previously published literature.^[^ ^44^ ^]^ The mice were sacrificed on day 21 post‐treatment. Maxilla samples and vital organs (heart, liver, spleen, lung, kidney) were collected and fixed for further analysis.

### 2.13 Micro‐CT Analysis

The fixed maxilla samples were scanned using high‐resolution micro‐CT. Mimics Research 21.0 software was used for 3D visualization of the periodontal bone tissues. Skyscan CT‐analyzer (CTAn) software was utilized to assess bone parameters, including bone volume fraction (BV/TV, %), trabecular thickness (Tb. Th, mm), trabecular number (Tb. N, 1/mm), and trabecular separation (Tb. Sp, mm).

### 2.14 Histological Observation and Immunofluorescence Staining

The fixed maxillary samples were decalcified for 4 weeks. Then the decalcified maxillary and other organs were dehydrated, embedded in paraffin, and sectioned successively at a thickness of 5.0 µm. Sections were stained with hematoxylin and eosin (H&E), and photographs were captured using a microscope. The expression levels of osteogenesis‐related proteins (OCN, EGFR, and RhoA) were analyzed by immunofluorescence staining. Cy3‐labeled goat anti‐rabbit IgG served as the secondary antibody. The immunofluorescence images were observed using a fluorescence microscope.

### 2.15 miRNA Expression Profiling

miRNA sequencing of NC‐EVs and EPO‐EVs was performed using the DNBSEQ platform (BGI). The sequencing libraries were constructed using single‐end 50 bp sequencing, generating 20 m reads of data per sample on the DNBSEQ™ technology platform. Differentially expressed miRNAs (DEMs) between NC‐EVs and EPO‐EVs were identified using |log_2_(Fold Change)|≥ 2 and Q‐value < 0.05 as filtering threshold. The raw sequencing data have been deposited in the NCBI GenBank under BioProject PRJNA1137900.

### 2.16 miRNA Target Prediction

The miRNA target prediction and analysis were conducted using the algorithms from RNAhybrid (https://bibiserv.cebitec.uni‐bielefeld.de/rnahybrid), miRanda (http://www.microrna.org/), and TargetScan (http://www.targetscan.org/). GO and KEGG pathway enrichment analysis of enriched predicted miRNA target genes were performed using OmicStudio tools. The STRING protein database was employed to establish the protein‐protein interaction (PPI) network for the target genes. Network visualization was performed using Cytoscape software.

### 2.17 Dual‐Luciferase Reporter Assay

The full‐length 3′ UTR of the wild‐type (WT) EGFR gene was cloned and inserted into the pmirGLO vector (Promega). Mutant (MUT) vectors were generated by site‐directed mutagenesis of the predicted miR‐5107‐5p binding site in the EGFR gene. HEK‐293 cells were seeded into 24‐well plates at a density of 1 × 10⁵ cells per well. When cells reached 60–70% confluence, HEK‐293 cells were co‐transfected with 1 µg of reporter gene vectors (WT‐EGFR or MUT‐EGFR) and 50 nm miR‐5107 mimic using Lipofectamine 3000 according to the manufacturer's instructions. Luciferase activity was measured using a dual luciferase reporter assay kit 48 h post‐transfection to compare the relative activity of Renilla and firefly luciferase.

### 2.18 Quantification and Statistical Analyses

All data are presented as the mean ± standard deviation (SD) from at least three independent experiments. Statistical analyses were conducted using GraphPad Prism 9. An independent two‐tailed Student's *t*‐test was used for comparing two groups, while one‐way ANOVA was applied for comparisons among more than two groups. A *p*‐value of less than 0.05 was considered statistically significant.

## 3 Results

### 3.1 Isolation and Characterization of NC‐EVs and EPO‐EVs

**Figure 1:**
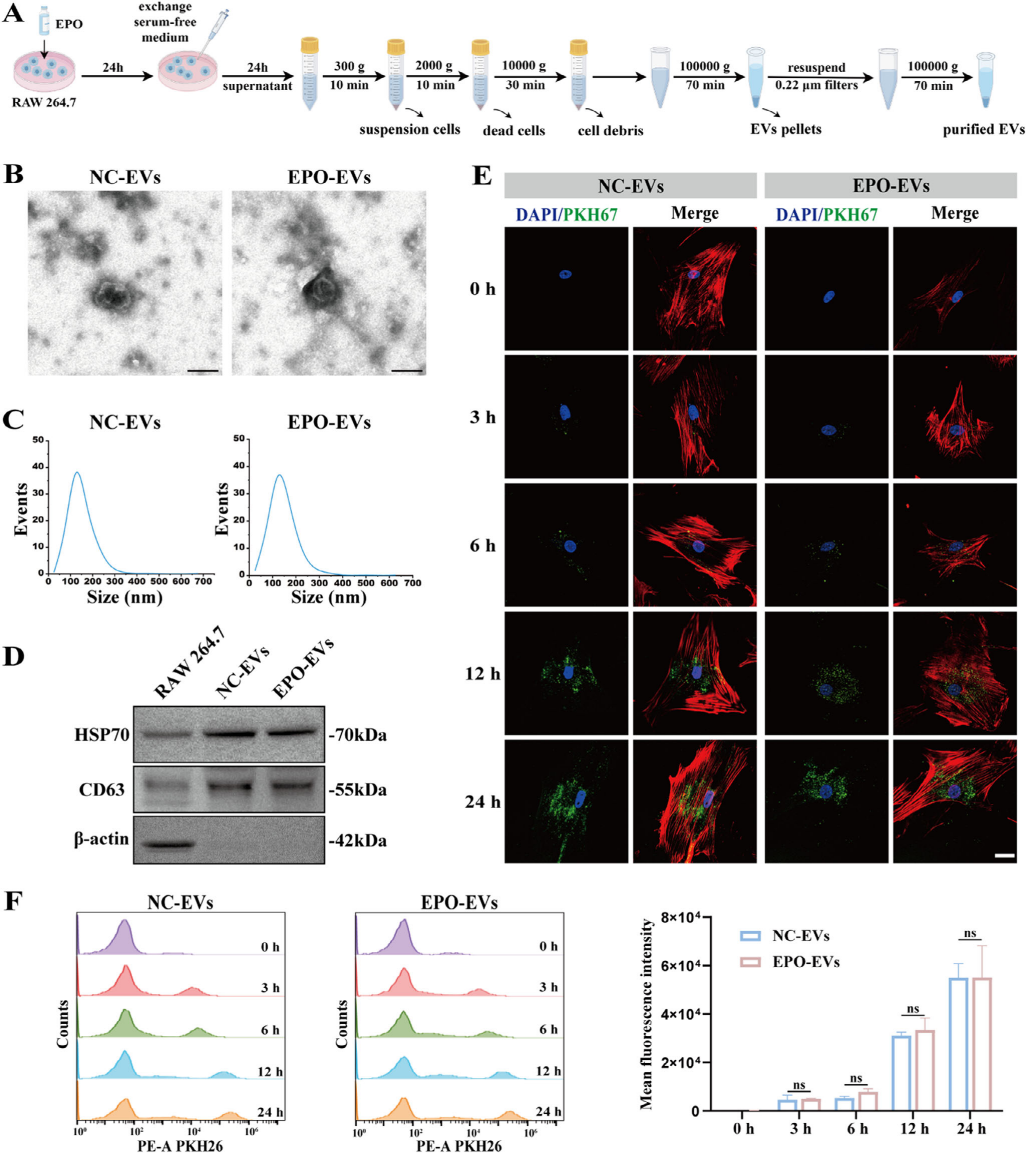
Isolation and characterization of NC‐EVs and EPO‐EVs. A) Flow chart of NC‐EVs and EPO‐EVs isolation (Created by Figdraw). B) Representative TEM images of NC‐EVs and EPO‐EVs. Scale bar = 100 nm. C) Analysis of the size distribution of NC‐EVs and EPO‐EVs. D) Western blot analysis of EVs surface marker proteins (HSP70, CD63) and cytosolic marker (β‐actin) in RAW 264.7 (Cells), NC‐EVs and EPO‐EVs (EVs). E) Representative images of the uptake of PKH67‐labeled EVs by mBMSCs at the indicated time points after coculture. Scale bar = 50 µm. F) Flow cytometry quantity analysis of mBMSCs which uptake the PKH26‐labeled EVs at the indicated time points after coculture (*n* = 3) (ns. No significant difference). The data are analyzed using independent two‐tailed Student's *t*‐test and presented as mean value ± SD.

We successfully isolated the EVs secreted by normal macrophages (NC‐EVs) and EPO‐stimulated macrophages (EPO‐EVs) through standard ultracentrifugation protocol (**Figure** **1**A). TEM revealed the typical bilayer membrane structure and saucer‐like shape of both NC‐EVs and EPO‐EVs, with no significant morphological differences (Figure 1B). NTA revealed comparable predominant particle sizes for both NC‐EVs and EPO‐EVs (mode: 130 ± 50.0 nm vs 131 ± 50.9 nm) (Figure 1C). Western blot analysis demonstrated the expression of the EV surface marker proteins HSP70 and CD63 in both NC‐EVs and EPO‐EVs. The cytoplasmic marker, β‐actin, was absent from the EVs lysates, confirming successful isolation (Figure 1D). To evaluate the functions of EVs in intercellular communication, delivering and releasing contents during bone regeneration, we assessed their uptake by mBMSCs. The EVs were labeled with PKH67 and incubated with mBMSCs. After 0, 3, 6, 12, and 24 h of incubation, confocal micrographs clearly revealed the endocytosis of PKH67‐labeled NC‐EVs and EPO‐EVs by mBMSCs. Time‐dependent increases of the PKH67‐labeled EVs (green) were observed around the nucleus (blue), with the cytoskeleton stained red (Figure 1E). We further quantitatively assessed the cellular uptake of EVs at the same indicated time points using flow cytometry analysis. Consistent with the confocal imaging, the results showed an increased fluorescence of mBMSCs after the addition of PKH26‐labeled EVs, with the most EV internalization observed between 12 and 24 h (Figure 1F). These results demonstrated that the isolated EVs exhibit typical morphological and molecular characteristics. Their consistent uptake by effector cells provides a solid foundation and therapeutic reference for subsequent functional validation experiments.

### 3.2 Characterization of EV‐Encapsulated CS/β‐GP/Gelatin Hydrogel

**Figure 2:**
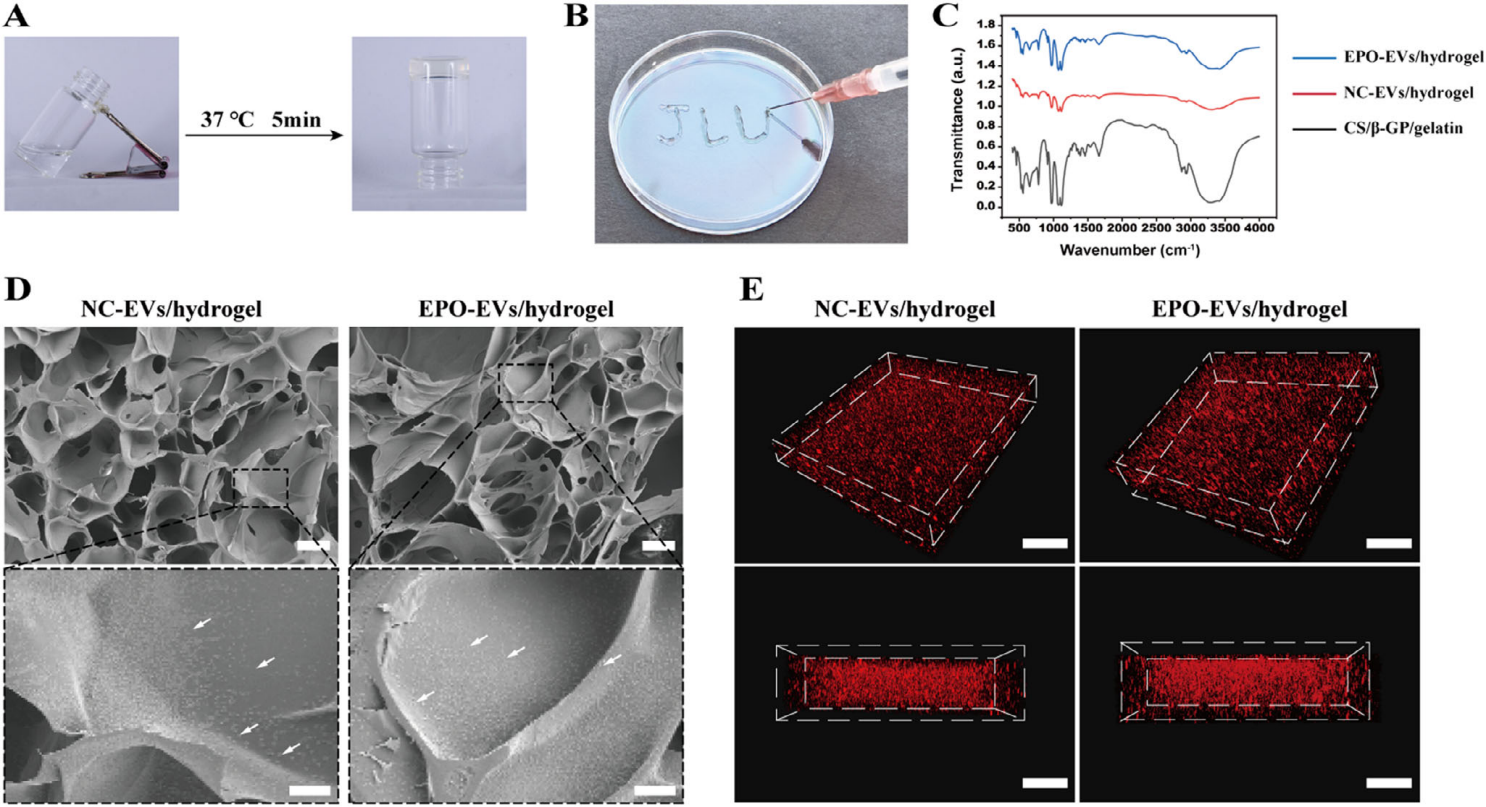
Characterization of EV‐encapsulated CS/β‐GP/gelatin hydrogel. A) Photographs of the freshly prepared pre‐gel solution and the hydrogel formed at body temperature. B) The pre‐gel solution was injected into irregular shapes. C) FTIR spectra of the lyophilized EV‐encapsulated hydrogels. D) Cross‐sectional morphologies of the hydrogels. Scale bar = 100 µm; Scale bar (Zoom) = 20 µm. The arrow denotes the EVs attached to the surface of the hydrogel. E) Representative 3D distribution of PKH26‐labeled EVs within the hydrogels. Scale bar = 100 µm.

Since CS/β‐GP/gelatin hydrogel has injectable and drugs sustained‐release properties,^[^ ^30^ ^]^ we incorporated EVs into these hydrogels to increase their stability and localization at injury sites. The mixed solution remained clear at room temperature and transitioned into a uniform gel state within 5 min at 37 °C (**Figure** **2**A). The gel injectability was not affected after the addition of EVs, and this pre‐gel solution was allowed for injection into non‐uniform sites prior to gelation (Figure 2B). The integration of the hydrogel components was confirmed by infrared spectroscopy, which demonstrated the successful synthesis of the CS/β‐GP/gelatin hydrogel (Figure S1, Supporting Information), hereafter referred to as “hydrogel”. Moreover, no significant differences in the composition were observed between the NC‐EVs‐encapsulated and EPO‐EVs‐encapsulated hydrogels (Figure 2C). The scanning electron microscopy (SEM) was employed to examine the cross‐sectional morphology of lyophilized hydrogels. The results revealed that the hydrogel structure was loose and porous (Figure S2, Supporting Information), suggesting that the network was well‐suited for EV loading and controlled release. Furthermore, the porous structure of the hydrogel was not affected by the encapsulation of EVs. Representative SEM images revealed that NC‐EVs and EPO‐EVs aggregated into irregular shapes and adhere to the hydrogel surface (Figure 2D), confirming the successful preparation of both NC‐EVs/hydrogel and EPO‐EVs/hydrogel. Additionally, the use of PKH26‐labeled EVs (NC‐EVs or EPO‐EVs) facilitated enhanced visualization of their distribution throughout the hydrogel via confocal laser scanning microscopy (CLSM). The 3D images of the cubic hydrogel demonstrated that either PKH26‐labeled NC‐EVs or EPO‐EVs were uniformly distributed within the hydrogel's loose and porous structure (Figure 2E). There results confirmed the successful preparation of the NC‐EVs/hydrogel and EPO‐EVs/hydrogel.

### 3.3 Biocompatibility of the Hydrogel and EVs Retention Evaluation within the Hydrogel

**Figure 3:**
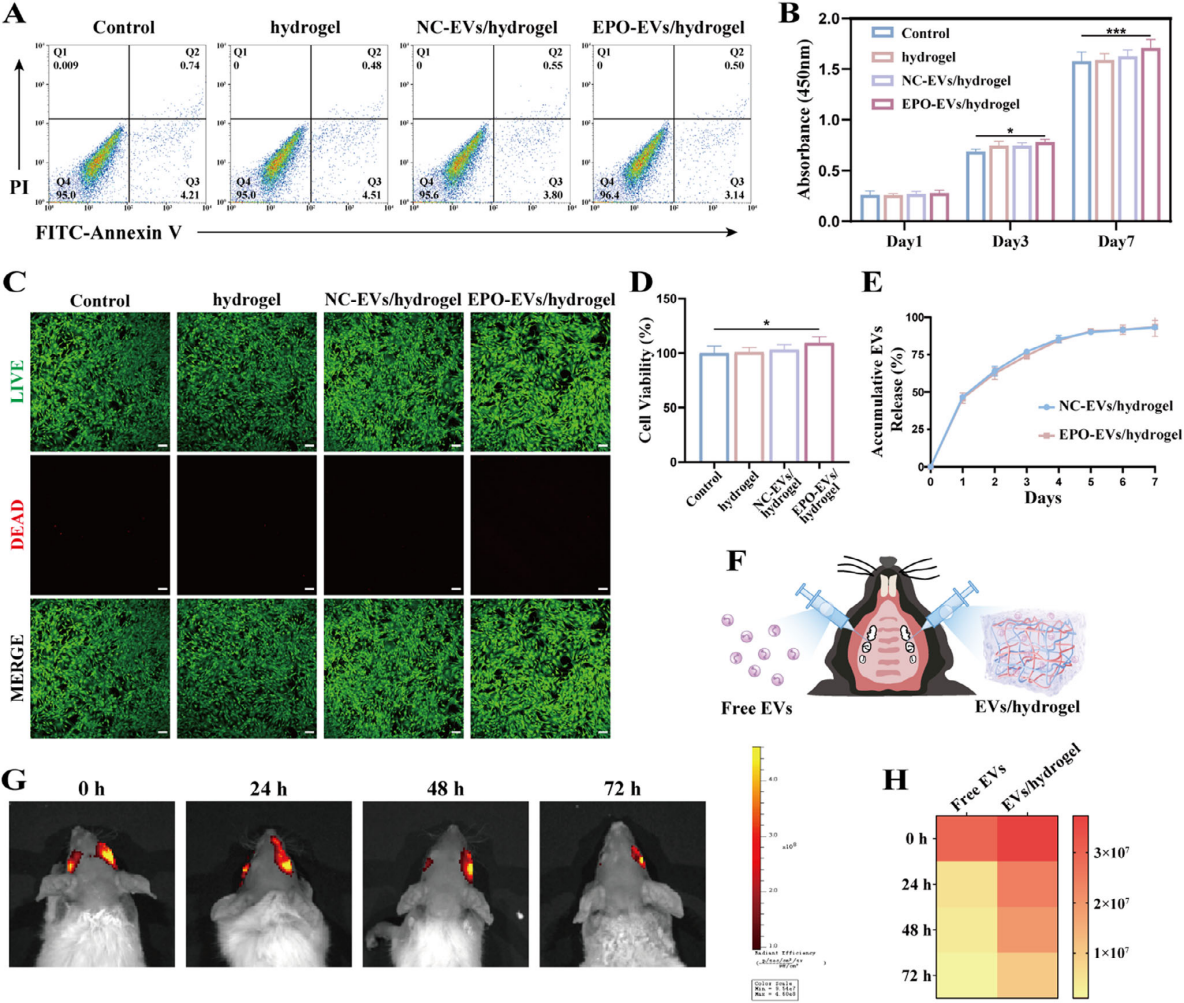
Biocompatibility of the hydrogel and EVs retention evaluation within the hydrogel. A) Flow cytometry analysis of apoptotic cell percentages after coculture with extracts from different hydrogels. B) CCK‐8 assay was performed after 1, 3, and 7 days of incubation (*n* = 3) (* *p* < 0.05, ** *p* < 0.01, *** *p* < 0.001). The data are analyzed using ANOVA and presented as mean value ± SD. C) Cell viability assay after 7 days of cell culture, green and red cells represent live and dead cells, respectively. Scale bar = 200 µm. D) Cell viability rate (*n* = 3) (* *p* < 0.05, ** *p* < 0.01, *** *p* < 0.001). The data are analyzed using ANOVA and presented as mean value ± SD. E) The release curves of EVs from the hydrogels. F) Schematic diagram of the mouse model to test the biodistribution of EVs following local periodontal injection. G) In vivo representative bioluminescence images of the retention of DiR‐labeled EPO‐EVs in free form and hydrogel form following local periodontal injection at different time points. H) The fluorescence intensity was quantified accordingly using heatmap.

A flow cytometry assay was used to assess cell apoptosis, evaluating the biological safety of the hydrogels. The results revealed that over 95% of mBMSCs remained in a healthy state after being cocultured with extracts from the hydrogel or EV‐encapsulated hydrogel (**Figure** **3**A). The CCK‐8 assay demonstrated that mBMSCs cocultured with extracts from EPO‐EVs/hydrogel exhibited significantly enhanced growth and proliferation compared to the hydrogel and NC‐EVs/hydrogel groups on day 7 (Figure 3B). Additionally, fluorescence staining of live/dead cells co‐cultured with various hydrogel extracts for 7 days demonstrated excellent cell viability across all four groups, with the EPO‐EVs/hydrogel group showing the most prominent green fluorescence (Figure 3C). Quantitative analysis further revealed that the EPO‐EVs/hydrogel group exhibited higher cell viability compared to the other three groups, indicating that the EPO‐EVs/hydrogel was more conducive to the growth and proliferation of mBMSCs (Figure 3D).

To assess the release ratio of EVs from the hydrogel, NC‐EVs/hydrogel and EPO‐EVs/hydrogel were immersed in PBS, and the EV concentration in the PBS was measured daily over the course of a week using the BCA assay. The results indicated that most of the EVs were released within this period. Due to the identical EV loading and preparation process, both types of EVs encapsulated in the hydrogels exhibited similar release behavior (Figure 3E). Besides evaluating the sustained release properties of EPO‐EVs/hydrogel in vitro, we also assess their retention through in vivo studies. DiR‐labeled free EPO‐EVs or EPO‐EVs/hydrogel was injected into the local periodontal pockets of mice, and the fluorescence signal intensity was tracked at various time points (Figure 3F). In vivo fluorescence imaging of live animals showed that the fluorescence intensity of free EPO‐EVs diminished over time, whereas EPO‐EVs/hydrogel retained strong fluorescence signals even after 72 h (Figure 3G). Heat map analysis showed a significant reduction in signal intensity for free EPO‐EVs at 24 h post‐injection. In contrast, the EPO‐EVs/hydrogel exhibited a slower decrease in signal intensity over the 72‐h period, suggesting that the hydrogel effectively extended the retention of EPO‐EVs in vivo and supported their sustained release from the hydrogel (Figure 3H).

The findings above confirmed that the porous structure of the hydrogel enabled efficient encapsulation and sustained release of EPO‐EVs, while also exhibiting excellent biocompatibility. These properties of the hydrogel matrix render it an ideal carrier platform for efficiently retaining EVs in irregular periodontal tissue defects.

### 3.4 EPO‐EVs Elevate Osteogenic Potential of mBMSCs in the Inflammatory Milieu In Vitro

**Figure 4:**
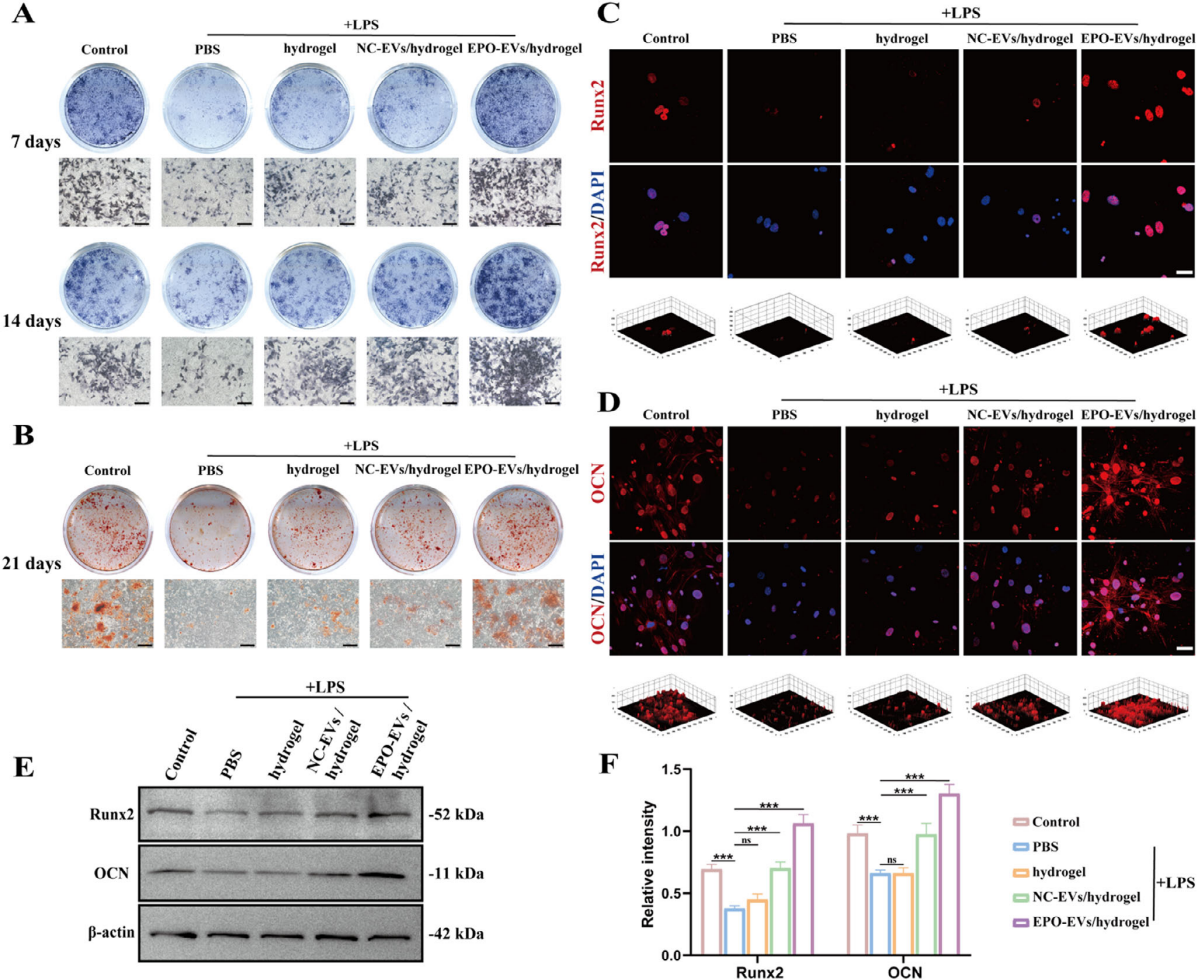
EPO‐EVs elevate osteogenic potential of mBMSCs in the inflammatory milieu in vitro. A) ALP staining images of mBMSCs on day 7and 14. Scale bar = 200 µm. B) Alizarin red staining images of mBMSCs on day 21. Scale bar = 200 µm. C) Immunofluorescence staining showed the expression of Runx2 on day 7. Scale bar = 50 µm. D) Immunofluorescence staining showed the expression of OCN on day 7. Scale bar = 50 µm. E) Western blot analysis of osteoblastic protein Runx2 and OCN in mBMSCs on day 7. F) Quantitative analysis of the relative intensity of WB bands in each group (*n* = 3) (* *p* < 0.05, ** *p* < 0.01, *** *p* < 0.001, ns. No significant difference). The data are analyzed using ANOVA and presented as mean value ± SD.

To investigate the impact of EPO‐EVs on mBMSCs in vitro under inflammatory conditions, we used LPS (1 µg mL⁻^1^) to induce inflammation and inhibit osteogenesis in mBMSCs. We observed that the transcript levels of osteogenic factors, including ALP, Col1α1, Runx2, and osteocalcin (OCN), were significantly decreased in mBMSCs exposed to an LPS‐induced chronic inflammatory microenvironment. However, the application of EPO‐EVs/hydrogel partially reversed the osteogenic inhibition caused by this inflammatory condition. In the group of inflammatory mBMSCs treated with EPO‐EVs/hydrogel, we observed varying degrees of improvement in the mRNA levels of ALP, Col1α1, Runx2, and OCN on days 7 and 14 (Figure S3, Supporting Information). Furthermore, no significant differences were observed between the hydrogel and PBS groups as the hydrogel components lacked growth factors or other anti‐inflammatory agents. ALP activity is an early‐phase osteogenic differentiation marker. As shown in the ALP staining images, the EPO‐EVs/hydrogel group exhibited significantly higher expression of ALP on both day 7 and 14 when compared to the LPS, hydrogel and NC‐EVs/hydrogel groups (**Figure** **4**A). Similarly, quantitative ALP assay confirmed a marked increase in ALP expression in mBMSCs treated with EPO‐EVs/hydrogel extracts for 7 and 14 days (Figure S4, Supporting Information). After incubation for 21 days, we used Alizarin Red S staining, a late‐stage osteoblastic marker, to evaluate the level of mineralization in mBMSCs. As shown in the representative images and the quantitative analysis of ARS staining, mineralization increased to varying degrees in all therapy groups compared to the PBS treatment group. Notably, the EPO‐EVs/hydrogel group demonstrated superior mineralization and osteogenic induction properties compared to the NC‐EVs/hydrogel and hydrogel groups (Figure 4B; Figure S5, Supporting Information). Additionally, immunofluorescence imaging further demonstrated an increase in Runx2 and OCN labeling in mBMSCs after 7 days of coculture with the extract of hydrogel, NC‐EVs/hydrogel or EPO‐EVs/hydrogel, with a more pronounced enhancement in the EPO‐EVs/hydrogel group (Figure 4C,D). Western blot bands and quantitative analysis of the gray values showed that the protein levels of OCN and Runx2 increased significantly on day 7 after EPO‐EVs/hydrogel treated (Figure 4E,F). Based on these findings, we conclude that EPO‐EVs effectively enhance the osteogenic activity of mBMSCs in inflammatory conditions, highlighting its significant potential for therapeutic applications in addressing inflammatory bone loss.

### 3.5 Analysis of the Differential Expression of miRNAs in NC‐EVs and EPO‐EVs

**Figure 5:**
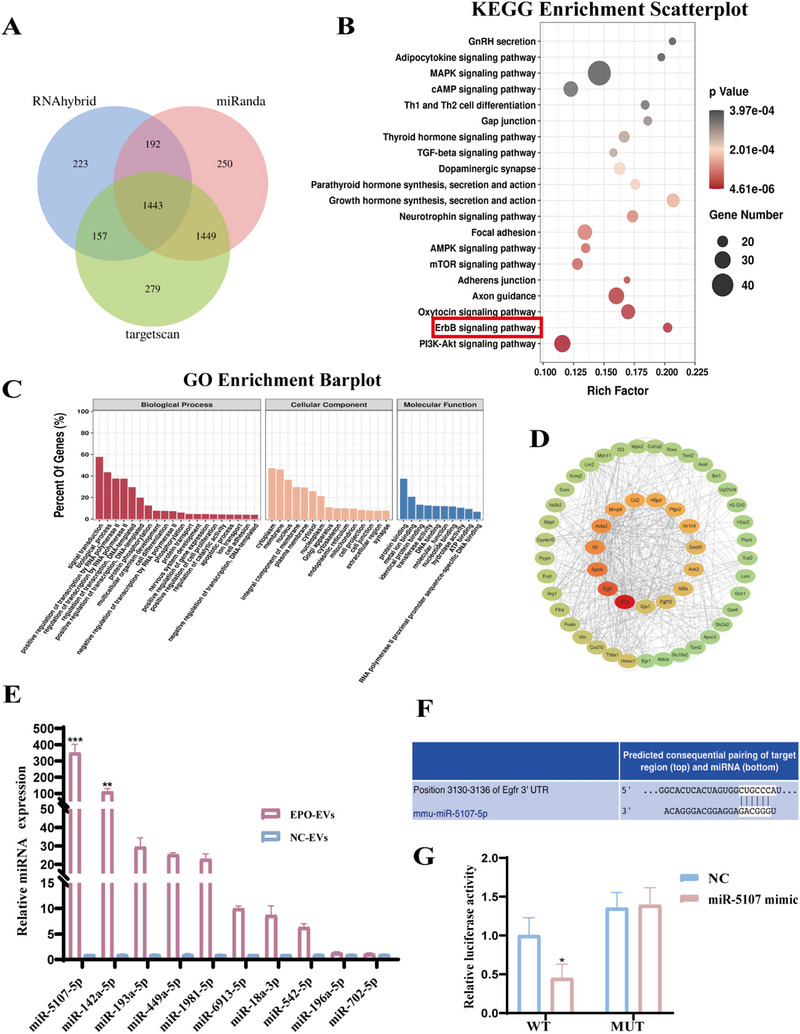
Analysis of the differential expression of miRNAs between NC‐EVs and EPO‐EVs. A) Three bioinformatics tools (TargetScan, RNAhybrid, and miRanda) were used to analyze genes targeted by top ten upregulated DEMs. B) KEGG enrichment analysis results were obtained by scatter plots. C) GO classification of the genes—biological process, cellular component, and molecular function. D) All genes targeted by top ten upregulated DEMs were shown in the PPI network. E) qPCR analysis showed the expression of miRNAs in NC‐EVs and EPO‐EVs (*n* = 3) (* *p* < 0.05, ** *p* < 0.01, *** *p* < 0.001). The data are analyzed using independent two‐tailed Student's *t*‐test and presented as mean value ± SD. F) The binding site between EGFR (position 3130–3136 of EGFR 3′ UTR) and miR‐5107‐5p as predicted by the TargetScan website. G) Luciferase activity in HEK‐293 cells transfected with EGFR‐Wt/Mut and mimic‐5107 or mimic‐NC (*n* = 3) (* *p* < 0.05). The data are analyzed using independent two‐tailed Student's *t*‐test and presented as mean value ± SD.

Since EVs play a key role in cellular communication by transferring miRNAs between cells, which can regulate post‐transcriptional gene expression in recipient cells.^[^ ^23^ ^]^ We hypothesized that the miRNAs in EPO‐EVs could influence osteogenic differentiation in an inflammatory environment by targeting genes involved in relevant signaling pathways. To explore this, we analyzed the miRNAs profiles of NC‐EVs and EPO‐EVs using the DNBSEQ platform. Our miRNAs sequencing analysis identified 230 overlapping miRNAs between NC‐EVs and EPO‐EVs (Figure S6a, Supporting Information). We discovered 79 DEMs (|log_2_(fold change)| ≥ 2 and Q value < 0.05) between the two EVs types, including 61 up‐regulated and 18 down‐regulated miRNAs in EPO‐EVs (Figure S6b, Supporting Information). For the top ten up‐regulated miRNAs in EPO‐EVs, we predicted their target genes using databases such as TargetScan, RNAhybrid, and miRanda, basing on the binding sites in the 3′ UTR. We then narrowed our investigation to the intersection of the target genes predicted by all three databases, identifying 1 443 highly probable target genes (**Figure** **5**A). KEGG pathway enrichment analysis showed that the targeted genes were associated with several signaling pathways, including the “ErbB signaling pathway”, “PI3K‐AKT signaling pathway”, “AMPK signaling pathway”, and “Focal adhesion”, indicating significant enrichment among these potential targets (Figure 5B). GO enrichment analysis showed that these target genes primarily contributed to “signal transduction”, “cell membrane composition”, “cytoplasmic composition”, and “protein binding” (Figure 5C). Additionally, PPI analysis illustrated a complex network of proteins with functional interactions (Figure 5D). Notably, the EGFR emerged as a central player in this network, acting as a key member of the cell membrane protein subfamily within the ErbB signaling pathway. Additionally, EV‐derived miRNAs can suppress gene expression by binding to the 3′ UTR of target mRNAs via base pairing. Bioinformatics algorithms identified EGFR as a target gene of miR‐5107‐5p, suggesting that miR‐5107‐5p can bind to the 3′ UTR of EGFR mRNA (Figure 5E). qPCR analysis confirmed a significant upregulation of miR‐5107‐5p in EPO‐EVs (Figure 5F), aligning with the miRNAs sequencing results. Moreover, the dual‐luciferase reporter assay demonstrated that mimic‐5107 downregulated the luciferase activity of the WT‐EGFR‐3′ UTR (Figure 5G). These findings confirm that miR‐5107‐5p directly binds to the 3′ UTR of EGFR mRNA, inhibiting its post‐transcriptional gene expression and downregulating EGFR levels.

### 3.6 Abundant miR‐5107‐5p in EPO‐EVs Promotes the Osteogenic Differentiation of Inflamed mBMSCs

**Figure 6:**
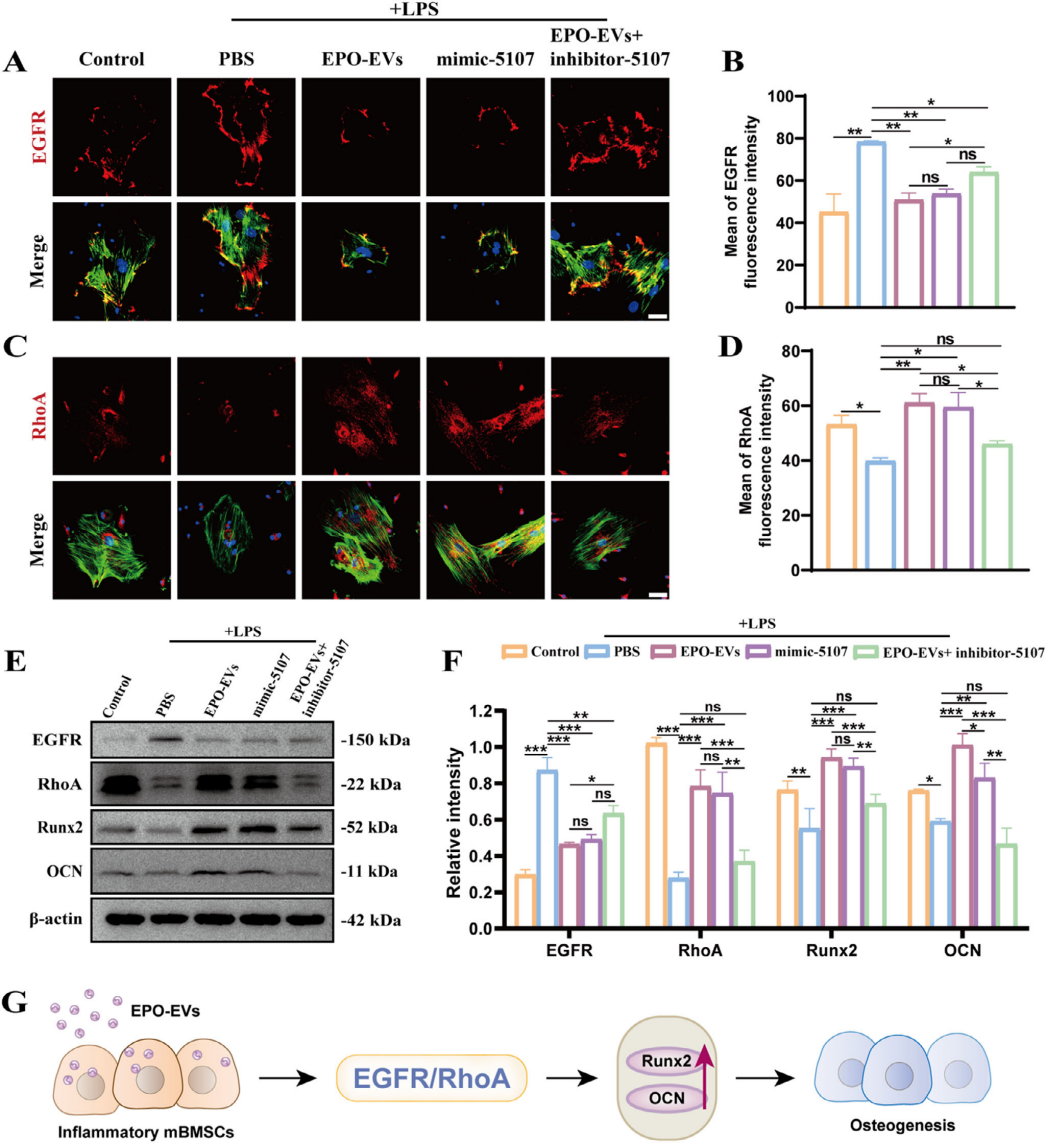
Abundant miR‐5107‐5p in EPO‐EVs promotes the osteogenic differentiation of inflamed mBMSCs. A) Immunofluorescence staining showed the expression of EGFR in the mBMSCs of each group. Nuclei were stained with DAPI. Scale bar = 50 µm. B) Comparative analysis of the fluorescence intensity of the expression of EGFR in the mBMSCs of each group (*n* = 3) (* *p* < 0.05, ** *p* < 0.01, *** *p* < 0.001, ns. No significant difference). The data are analyzed using ANOVA and presented as mean value ± SD. C) Immunofluorescence staining showed the expression of RhoA in the mBMSCs of each group. Nuclei were stained with DAPI. Scale bar = 50 µm. D) Comparative analysis of the fluorescence intensity of the expression of RhoA in the mBMSCs of each group (*n* = 3) (* *p* < 0.05, ** *p* < 0.01, *** *p* < 0.001, ns. No significant difference). The data are analyzed using ANOVA and presented as mean value ± SD. E) Western blot analysis of EGFR, RhoA, Runx2, and OCN in mBMSCs from each group. F) Quantitative analysis of the relative intensity of WB bands in each group (*n* = 3) (* *p* < 0.05, ** *p* < 0.01, *** *p* < 0.001, ns. No significant difference). The data are analyzed using ANOVA and presented as mean value ± SD. G) Schematic diagram of EPO‐EVs‐induced osteogenic differentiation of mBMSCs.

To further explore whether miR‐5107‐5p regulates the osteogenesis of inflammatory mBMSCs by suppressing EGFR, we transfected the miR‐5107‐5p‐specific inhibitor (inhibitor‐5107) or the specific activator (mimic‐5107) into the mBMSCs which were co‐cultured with EPO‐EVs. The represent IF imaging of mBMSCs demonstrated that both EPO‐EVs and mimic‐5107 treatments reduced EGFR fluorescence signals, which were significantly increased in the inflammatory milieu (**Figure** **6**A). Quantitative fluorescence intensity analysis revealed significantly lower EGFR levels after EPO‐EVs or mimic‐5107 treatment compared to LPS treatment alone (Figure 6B). RhoA has been shown to influence osteogenic differentiation and morphological changes in MSCs.^[^ ^45^, ^46^ ^]^ However, it is unclear whether the miR‐5107‐5p / EGFR axis can regulate the expression of RhoA. Conversely to the EGFR, the fluorescence intensity of RhoA was significantly downregulated following inflammatory stimulation but showed a marked increase after EPO‐EVs or mimic‐5107 therapy (Figure 6C,D). Nevertheless, the effects of EGFR upregulation and RhoA downregulation were reversed upon the addition of inhibitor‐5107. Western blot images and quantitative analysis further confirmed that EGFR protein expression was elevated under inflammatory conditions, but treatment with EPO‐EVs or mimic‐5107 suppressed this process while simultaneously upregulating RhoA protein expression. Meanwhile, the osteogenic‐related genes Runx2 and OCN showed a significant increase in inflamed mBMSCs treated with EPO‐EVs or mimic‐5107 whereas this osteogenic induction effect was suppressed by the specific inhibitor, inhibitor‐5107 (Figure 6E,F). These findings collectively suggest that the high levels of miR‐5107‐5p in EPO‐EVs enhance the potential for bone regeneration in an inflammatory environment through targeting EGFR and upregulating RhoA (Figure 6G).

### 3.7 EPO‐EVs Alleviate Inflammatory Alveolar Bone Loss in Periodontitis Mice

**Figure 7:**
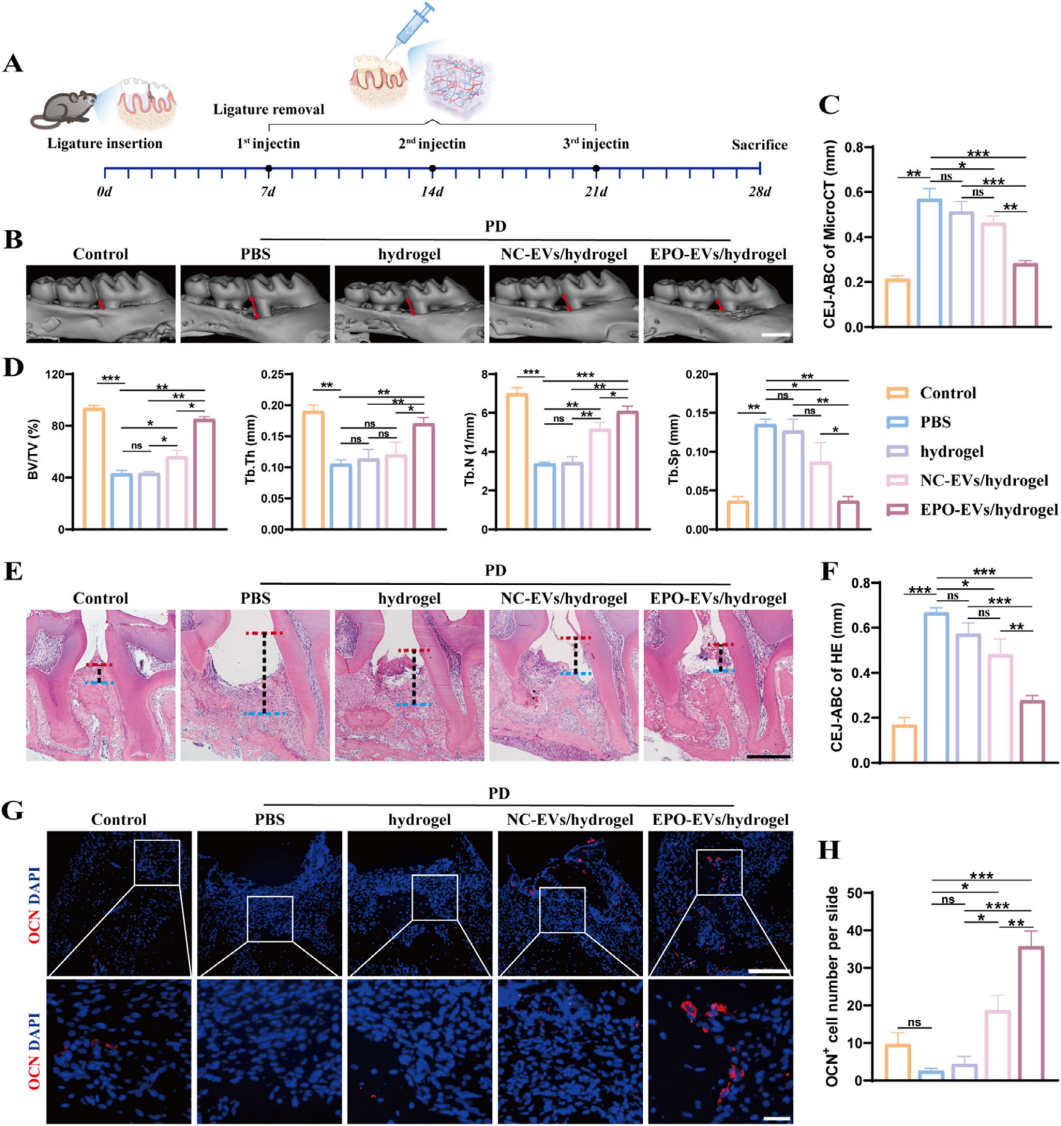
EPO‐EVs alleviate inflammatory alveolar bone loss in periodontitis mice. A) Schematic illustration for ligature‐induced periodontitis mouse model construction and the time point of local periodontal injection. B) 3D reconstruction of the maxillary samples in each group by micro‐CT scan. The red double arrows extend from the CEJ to the apex of the ABC. Scale bar = 1 mm. C) Quantification analysis of the distance from CEJ to ABC in each group as determined by micro‐CT (*n* = 5) (* *p* < 0.05, ** *p* < 0.01, *** *p* < 0.001, ns. No significant difference). The data are analyzed using ANOVA and presented as mean value ± SD. D) Quantitative analysis of BV/TV, Tb. Th, Tb. N and Tb. Sp. (*n* = 5) (* *p* < 0.05, ** *p* < 0.01, *** *p* < 0.001, ns. No significant difference). The data are analyzed using ANOVA and presented as mean value ± SD. E) Histological H&E staining of the periodontium in each group. The vertical lines extend from the CEJ to the ABC. Scale bar = 100 µm. F) Quantification analysis of the distance from CEJ to ABC in each group as determined by H&E staining (*n* = 5) (* *p* < 0.05, ** *p* < 0.01, *** *p* < 0.001, ns. No significant difference). The data are analyzed using ANOVA and presented as mean value ± SD. G) Immunofluorescence staining showed the expression of OCN in the periodontium of each group. Nuclei were stained with DAPI. Scale bar = 100 µm; Scale bar (Zoom) = 20 µm. H) Statistical analysis of the IF staining of periodontal OCN^+^ cells in each group (*n* = 5). The number of OCN^+^ cells was quantified for each microscope field of view (* *p* < 0.05, ** *p* < 0.01, *** *p* < 0.001, ns. No significant difference). The data are analyzed using ANOVA and presented as mean value ± SD.

Subsequently, we established a mouse periodontitis model to evaluate the therapeutic effects of EPO‐EVs on inflammatory bone loss in vivo (**Figure** **7**A). The histological sections of heart, liver, spleen, lung and kidney tissue showed no obvious abnormalities in EPO‐EVs/hydrogel, NC‐EVs/hydrogel, hydrogel, PBS and control groups (Figure S7, Supporting Information). Three‐dimensional micro‐CT reconstructions revealed significant changes in the alveolar bone between the first and second molars of the mouse following various treatments. Alveolar bone loss was assessed by measuring the distance from the cemento‐enamel junction (CEJ) to the apex of the alveolar bone crest (ABC). Micro‐CT analysis showed that the CEJ‐ABC distance significantly increased in the periodontitis (PD) model groups after silk ligation, compared to the healthy control group, confirming the successful establishment of the periodontitis model. While the EVs carrier hydrogel alone showed no therapeutic efficacy, the CEJ‐ABC distance was significantly reduced in the EPO‐EVs/hydrogel group (Figure 7B,C). Furthermore, in comparison to the NC‐EVs/hydrogel group, the EPO‐EVs/hydrogel group demonstrated more significant enhancements in critical metrics of bone regeneration. These improvements included increased BV/TV, Tb.Th, and Tb.N, along with decreased Tb.Sp, suggesting that EPO‐EVs have a significantly stronger ability to promote bone regeneration compared to NC‐EVs (Figure 7D). Additionally, H&E staining illustrated the morphology of the periodontium between the first and second molars. In the control group, the junctional epithelium remained attached to the CEJ, and the ABC showed no signs of resorption. In contrast, the PBS group with ligation exhibited epithelial migration toward the root, bone structure breakdown, and increased inflammatory infiltration. This damage was notably mitigated following EPO‐EVs/hydrogel treatment (Figure 7E). The CEJ‐ABC distance was significantly shorter in the EPO‐EVs/hydrogel group compared to the PBS, hydrogel, and NC‐EVs/hydrogel groups (Figure 7F). Moreover, we performed immunofluorescence staining for osteocalcin (OCN) to evaluate osteogenic expression during bone formation. OCN plays a critical role in bone mineralization and density by binding calcium and promoting its incorporation into the bone matrix. The representative IF images showed that OCN expression was reduced in the periodontitis (PD) model and significantly increased after 4‐week‐EPO‐EVs/hydrogel treatment (Figure 7G). Quantitative analysis revealed a significantly higher number of OCN+ cells in the periodontal tissues of the EPO‐EVs/hydrogel group compared to the PBS, hydrogel, and NC‐EVs/hydrogel groups (Figure 7H). Collectively, these results suggest that EPO‐EVs/hydrogel effectively alleviated the destruction of alveolar bone in the experimental periodontitis mouse model.

### 3.8 miR‐5107‐5p in EPO‐EVs Alleviates Inflammatory Bone Loss In Vivo through EGFR/RhoA Axis

**Figure 8:**
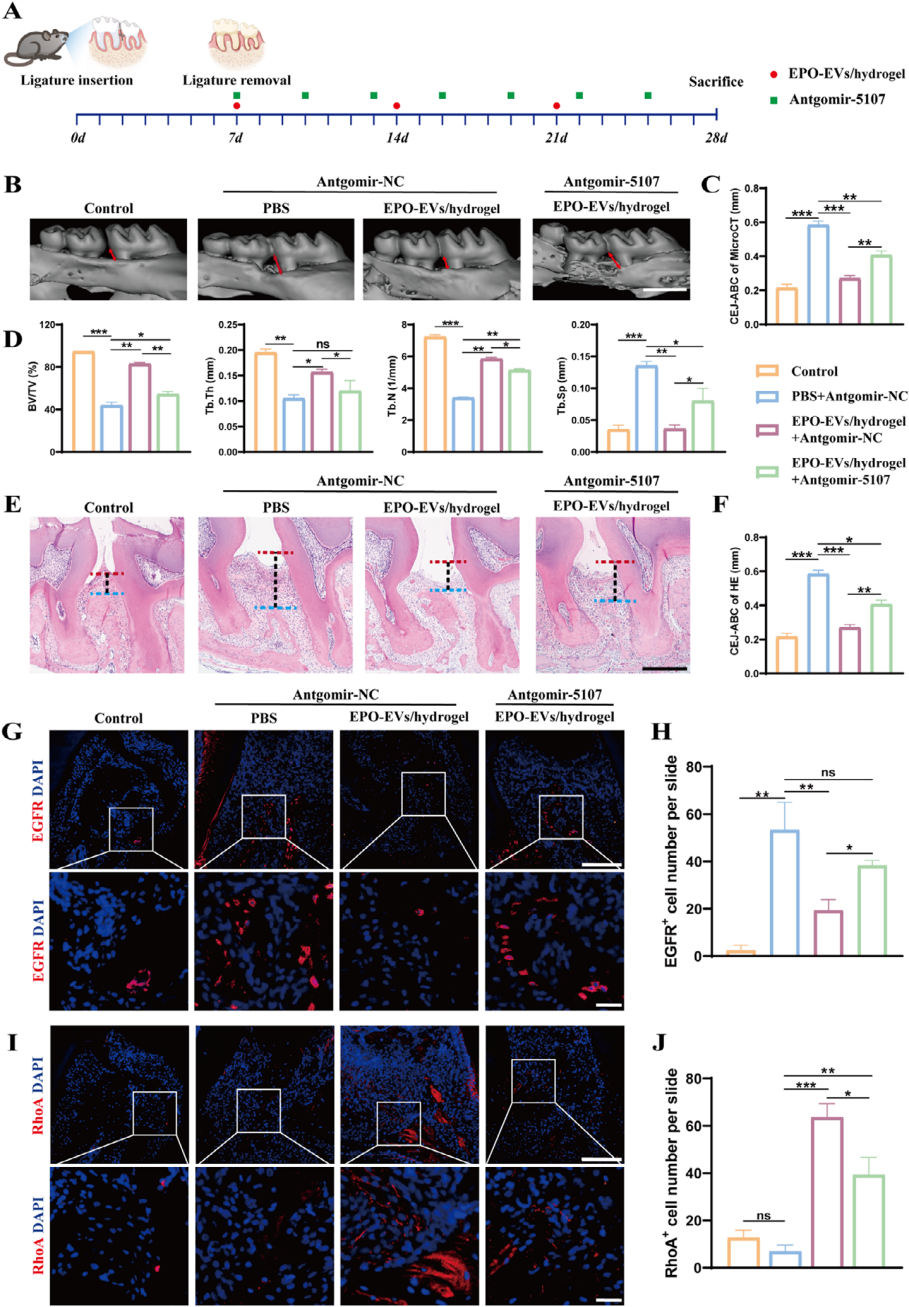
miR‐5107‐5p in EPO‐EVs alleviates inflammatory bone loss in vivo through EGFR/RhoA axis. A) Schematic diagram of the ligature‐induced periodontitis mice model and the time point of local periodontal injection. B) 3D reconstruction of the maxillary samples in each group by micro‐CT scan. The red double arrows extend from the CEJ to the apex of the ABC. Scale bar = 1 mm. C) Quantification analysis of the distance from CEJ to ABC in each group as determined by micro‐CT (*n* = 5) (* *p* < 0.05, ** *p* < 0.01, *** *p* < 0.001, ns. No significant difference). The data are analyzed using ANOVA and presented as mean value ± SD. D) Quantitative analysis of BV/TV, Tb. Th, Tb. N and Tb. Sp. (*n* = 5) (* *p* < 0.05, ** *p* < 0.01, *** *p* < 0.001, ns. No significant difference). The data are analyzed using ANOVA and presented as mean value ± SD. E) Histological H&E staining of the periodontium in each group. The vertical lines extend from the CEJ to the ABC. Scale bar = 100 µm. F) Quantification analysis of the distance from CEJ to ABC in each group as determined by H&E staining (*n* = 5) (* *p* < 0.05, ** *p* < 0.01, *** *p* < 0.001, ns. No significant difference). The data are analyzed using ANOVA and presented as mean value ± SD. G) Immunofluorescence staining showed the expression of EGFR in the periodontium of each group. Nuclei were stained with DAPI. Scale bar = 100 µm; Scale bar (Zoom) = 20 µm. H) Statistical analysis of the IF staining of periodontal EGFR+ cells in each group (*n* = 5). The number of EGFR+ cells was quantified for each microscope field of view (* *p* < 0.05, ** *p* < 0.01, *** *p* < 0.001, ns. No significant difference). The data are analyzed using ANOVA and presented as mean value ± SD. I) Immunofluorescence staining showed the expression of RhoA in the periodontium of each group. Nuclei were stained with DAPI. Scale bar = 100 µm; Scale bar (Zoom) = 20 µm. J) Statistical analysis of the IF staining of periodontal RhoA+ cells in each group (*n* = 5). The number of RhoA+ cells was quantified for each microscope field of view (* *p* < 0.05, ** *p* < 0.01, *** *p* < 0.001, ns. No significant difference). The data are analyzed using ANOVA and presented as mean value ± SD.

To investigate whether miR‐5107‐5p suppresses EGFR expression and contributes to EPO‐EVs‐mediated bone regeneration under inflammatory conditions in vivo, we employed Antagomir‐5107 as miR‐5107‐5p specific inhibitor in the periodontitis mice model every 3 days after ligature removal (**Figure** **8**A). Micro‐CT analysis revealed that Antagomir‐5107 notably diminished the bone regeneration effect mediated by EPO‐EVs. Compared to the EPO‐EVs/hydrogel group, the EPO‐EVs/hydrogel + Antagomir‐5107 group exhibited greater alveolar bone loss (Figure 8B,C), along with reduced levels of BV/TV, Tb. Th, and Tb. N, and higher levels of Tb. Sp (Figure 8D). Moreover, H&E staining showed a longer CEJ‐ABC distance, thinner epithelial layers of periodontal tissues, more infiltrating inflammatory cells, and less newly formed alveolar bone in the EPO‐EVs/hydrogel + Antagomir‐5107 group compared to the EPO‐EVs/hydrogel group (Figure 8E,F). IF imaging and statistical analysis indicated that EGFR levels, which were elevated in the periodontal tissues of PD mice, were downregulated by EPO‐EVs treatment but restored with Antagomir‐5107 administration (Figure 8G,H). Conversely, RhoA expression levels exhibited an inverse pattern to EGFR, showing a significant increase after EPO‐EVs/hydrogel treatment, which was attenuated by Antagomir‐5107 (Figure 8I,J). These results above support that miR‐5107‐5p in EPO‐EVs promotes alveolar bone regeneration in vivo mouse periodontitis model via the EGFR / RhoA axis.

## 4 Discussion

Current therapeutic approaches for periodontitis primarily focus on plaque control.^[^ ^4^, ^5^ ^]^ Although microenvironment regulation holds promise for periodontal tissue regeneration,^[^ ^47^ ^]^ it is insufficient in restoring the osteogenic function of endogenous BMSCs that have been impaired by inflammation. This limitation results in the continued loss of periodontal bone tissue, posing a significant challenge in the treatment of periodontitis.

Recently, miRNA‐based epigenetic therapy has gained significant attention due to its promising therapeutic potential. As small nucleotide chains consisting of 21–23 bases, miRNAs can be difficult to maintain its biological activity when used directly. These challenges include rapid degradation by nucleases, limited cellular uptake, and the risk of off‐target effects, which may induce immune responses and trigger reticuloendothelial clearance.^[^ ^23^, ^48^ ^]^ To address these issues, miRNAs are often encapsulated in liposomes or delivered to the target site as part of EVs, ensuring both their stability and effectiveness.^[^ ^49^, ^50^ ^]^ EVs, characterized by their excellent biocompatibility and low immunogenicity, can evade rapid clearance and are efficiently absorbed by recipient cells. Moreover, they can be produced and stored in large quantities, making them well‐suited for clinical applications.^[^ ^21^ ^]^ Studies have demonstrated that factors such as tannic acid, erythropoietin (EPO), or environmental conditions like hypoxia can influence the type and abundance of miRNAs in parent cells.^[^ ^27^, ^34^, ^51^ ^]^ These nucleotide chains are selectively enriched in EVs via a “sponge effect,” enabling precise regulation of gene expression while minimizing the side effects typically associated with direct drug administration.

Erythropoietin (EPO), a glycoprotein hormone, has been shown to possess diverse biological functions, including anti‐inflammatory, hematopoietic, and bone‐regenerative effects.^[^ ^31^, ^32^, ^33^ ^]^ However, its anti‐inflammatory efficacy is relatively limited unless used in combination with interleukin‐4 (IL‐4).^[^ ^52^ ^]^ Additionally, the osteogenic potential of EPO often requires long‐term, high‐dose administration, which carries significant risks of thrombosis and cardiovascular complications, as shown in mouse models and meta‐analyses of randomized clinical trials.^[^ ^53^, ^54^, ^55^ ^]^ As a result, the standalone application of EPO has demonstrated limited therapeutic efficacy, necessitating combination therapies to enhance its effects.^[^ ^56^ ^]^ These limitations pose challenges for the clinical translation of EPO therapy, underscoring the need for alternative approaches to maximize its therapeutic potential while minimizing risks. In our study, we successfully isolated EVs from normal control macrophages (NC‐EVs) and EPO‐stimulated macrophages (EPO‐EVs). The efficient uptake of both NC‐EVs and EPO‐EVs by mBMSCs suggests their significant potential to enhance osteogenesis in recipient cells. For the first time, we demonstrated that EPO‐EVs exert positive effects on bone regeneration within an inflammatory environment, as shown in both in vitro experiments and a mouse periodontitis model in vivo.

A key challenge for the clinical application of EVs lies in their poor stability and limited delivery efficiency. Hydrogels with 3D network structures provide an ideal platform for stabilizing EVs and modulating their release behavior. Chitosan‐based hydrogels, in particular, offer superior mechanical strength and resistance to degradation compared to similar materials, while maintaining excellent mobility and biocompatibility.^[^ ^37^, ^38^ ^]^ These properties enhance the therapeutic duration of EV‐based treatments. Additionally, the positive charges on chitosan interact with the negatively charged phospholipid membranes of EVs, offering significant potential for EV delivery applications.^[^ ^39^ ^]^ In our research, EPO‐EVs were encapsulated within a CS/β‐GP/gelatin hydrogel, which allowed for smooth penetration into periodontal pockets and stabilization for over 72 h. This system facilitated the release of small nucleotide chains from EVs into recipient cells, facilitating cell‐to‐cell communication and enhancing therapeutic effects.

Given the similarity between NC‐EVs and EPO‐EVs in their basic characteristics and cellular uptake patterns, we focused on potential functional differences in their contents to further explore the mechanisms underlying the promotion of osteogenic differentiation. EV‐derived miRNAs, lncRNAs, and DNA fragments can influence the biological functions of recipient cells through interacting with cell surface receptors or entering the nucleus.^[^ ^57^ ^]^ Specifically, miRNAs suppress gene expression by binding to the 3′ UTR of target mRNAs, leading to translational repression in recipient cells.^[^ ^23^ ^]^ Previous study has demonstrated that EPO primarily upregulates miRNAs expression in stem cells, enhancing their protective effects following kidney injury.^[^ ^34^ ^]^ Building on these evidences, we hypothesized that EVs from EPO‐stimulated macrophages, like most EVs, would carry an abundant miRNAs cargo capable of influencing the osteogenic process in mBMSCs. Herein, we investigated the fundamental mechanisms of epigenetic regulation mediated by specific miRNAs encapsulated in EPO‐EVs. Consistent with our hypothesis, miRNAs sequencing analysis revealed 79 DEMs in EVs derived from EPO‐stimulated macrophages, with 61 upregulated and 18 downregulated (filtered by |log_2_(fold change)| ≥ 2, Q value < 0.05). Target gene prediction of the top ten upregulated miRNAs showed enrichment in signaling pathways associated with osteogenesis and anti‐inflammatory processes. Among these, miR‐5107‐5p emerged as the most significantly upregulated miRNA, shown to target EGFR mRNA, thereby reducing EGFR protein expression levels.

Next, we focused on EGFR as a potential target due to its crucial role in skeletal homeostasis as a key component of the ErbB signaling pathway, primarily exerting an anabolic effect. The activation of EGFR in experimental LPS‐induced periodontitis models has been associated with negative regulation of osteoblastic differentiation.^[^ ^12^ ^]^ This activation by bacterial components highlights the complex relationship between inflammation and bone healing, suggesting that EGFR signaling in inflammatory BMSCs may impact their osteogenic potential and response to bone injury or disease. Additionally, previous research by Bi et al. demonstrated that inhibiting EGFR signaling significantly reduced inflammatory bone loss in a periodontal disease (PD) model.^[^ ^15^ ^]^ These findings underscore the role of EGFR in bone biology and its potential as a therapeutic target in inflammatory conditions. Our study identifies miR‐5107‐5p, enriched in EPO‐EVs, as a potential regulator of osteogenic processes in mBMSCs under inflammatory conditions. This regulation occurs primarily through the suppression of EGFR expression, offering a novel mechanism by which EPO‐EVs promote bone regeneration.

Recent studies suggest that EGFR signaling may play a role in Rho protein‐mediated remodeling of the actin cytoskeleton.^[^ ^58^, ^59^ ^]^ During osteogenic differentiation, cytoskeleton construction is a pivotal step in the development of osteocyte morphology. As a downstream effector of EGFR signaling, RhoA is crucial for modulating osteogenic differentiation, primarily by regulating cytoskeletal dynamics and cellular signaling pathways for osteogenic differentiation.^[^ ^46^, ^49^ ^]^ Given that, critical cellular functions, such as intracellular organization and membrane trafficking, rely on actin cytoskeleton dynamics. Modulating RhoA activity is essential for counteracting pathological conditions involving abnormal cytoskeletal remodeling. This process, in turn, promotes osteogenesis in mesenchymal stem cells.^[^ ^60^, ^61^ ^]^ Our findings indicate that the inflammatory environment impairs RhoA activity through EGFR activation, disrupting actin dynamics and reducing osteoblast differentiation. The data demonstrate that the addition of mimic‐5107 or EPO‐EVs effectively targets EGFR and further enhances RhoA activity, thereby alleviating inflammatory bone loss in LPS‐induced mBMSCs and promoting alveolar bone development in periodontitis mouse models. Conversely, treatment with inhibitor‐5107 counteracted these effects, further supporting the role of miR‐5107‐5p in mediating osteogenesis via EGFR/RhoA axis under inflammatory conditions.

In summary, our findings demonstrate that EPO‐EVs, highly enriched with miR‐5107‐5p, partially mitigate inflammatory bone loss in periodontitis through the EGFR/RhoA axis, suggesting a potential therapeutic target for periodontitis treatment. This study, primarily aimed at restoring stem cell function within an inflammatory microenvironment, also provides new insights into the use of EPO‐stimulated macrophage‐derived EVs for epigenetic modulation of recipient cells. Meanwhile, these findings have significant implications for stem cell‐mediated regeneration and repair of periodontal tissues under inflammatory conditions. However, this study has certain limitations. The mechanisms underlying EPO‐induced miR‐5107‐5p upregulation require further elucidation, and the current validation is restricted to murine models. Additional validation across different species is necessary to advance the clinical translation of this approach. Moreover, compared to conventional treatment options such as SRP, or even stem cell therapies, EPO‐EVs therapy might face the challenges in terms of long‐term efficacy, cost, and regulatory approval.

## Conflict of Interest

The authors declare no conflict of interest.

## Author Contributions

Shuchen Liu dealt with conceptualization, data curation, formal analysis, investigation, methodology, software, validation, visualization, and writing–original draft. Zhuoran Wang carried out data curation, investigation, methodology, software, validation, visualization, and writing–original draft. Yuhuan Li dealt with formal analysis, investigation, methodology, and writing–review & editing. Ziyi Pan dealt with methodology. Lei Huang conducted methodology. Jing Cui performed investigation. Xue Zhang carried out investigation. Mingxi Yang carried out methodology. Yuan Zhang dealt with resources and supervision. Daowei Li conducted project administration, resources, supervision, and writing–review & editing. Hongchen Sun carried out project administration, resources, and supervision.

## Supporting information



Supporting Information

## Data Availability

The data that support the findings of this study are available from the corresponding author upon reasonable request.
