# Supplementary material for: Erythropoietin‐Stimulated Macrophage‐Derived Extracellular Vesicles in Chitosan Hydrogel Rescue BMSCs Fate by Targeting EGFR to Alleviate Inflammatory Bone Loss in Periodontitis
Source: Adv Sci (Weinh). 2025 Apr 28;12(23):2500554. doi: 10.1002/advs.202500554 (PMC12199399; doi:10.1002/advs.202500554)
Supplement: Supplementary file 1 — Supporting Information [file ADVS-12-2500554-s001.docx]

Supplementary Information

**Erythropoietin-Stimulated Macrophage-Derived Extracellular Vesicles in Chitosan Hydrogel Rescue BMSCs Fate by Targeting EGFR to Alleviate Inflammatory Bone Loss in Periodontitis**

Shuchen Liu#^1^, Zhuoran Wang#^1^, Yuhuan Li ^3,4^, Ziyi Pan ^1^, Lei Huang ^1^, Jing Cui ^1^, Xue Zhang ^1^, Mingxi Yang ^4^, Yuan Zhang*^,2^, Daowei Li *^,1^, Hongchen Sun *^,1^

1. Jilin Provincial Key Laboratory of Tooth Development and Bone Remodeling, School and Hospital of Stomatology, Jilin University, 1500 Qinghua Road, Changchun, China.
2. Department of Anesthesiology, The First Hospital of Jilin University, 71 Xinmin Street, Changchun, China.
3. Department of Surgery, Experimental Surgery, CCM, CVK, Charité - Universitätsmedizin Berlin, Freie Universität Berlin and Humboldt-Universität zu Berlin, Berlin, Germany.
4. Department of Orthopedics, China-Japan Union Hospital of Jilin University, Changchun, China.
5. State Key Laboratory of Supramolecular Structure and Materials, College of Chemistry, Jilin University, Changchun, China.

*Corresponding authors: hcsun@jlu.edu.cn (Hongchen Sun); jluldw@jlu.edu.cn (Daowei Li); zhangyuansmile@jlu.edu.cn (Yuan Zhang)


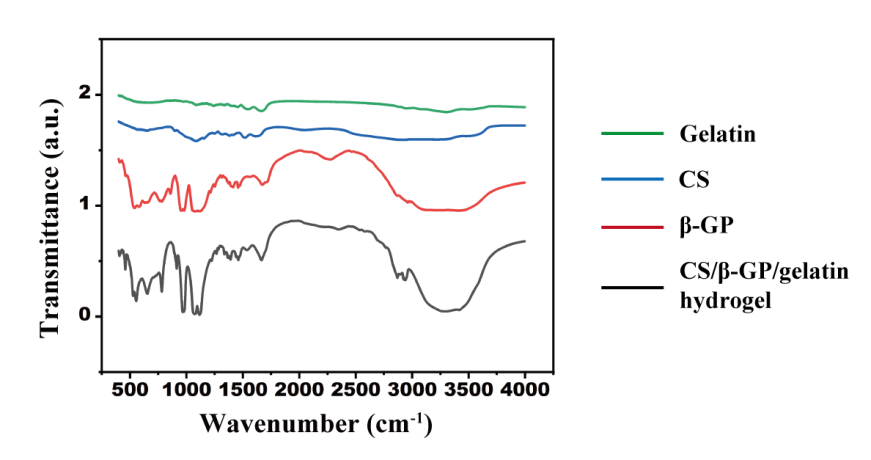


**Figure S1.** FTIR spectra of the lyophilized CS/β-GP/gelatin hydrogel.

**
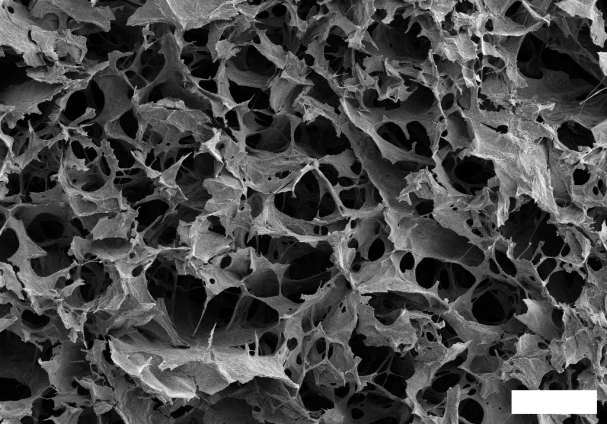
**

**Figure S2.** Cross-sectional morphologies of the CS/β-GP/gelatin hydrogel. Scale bar = 200 μm.

**
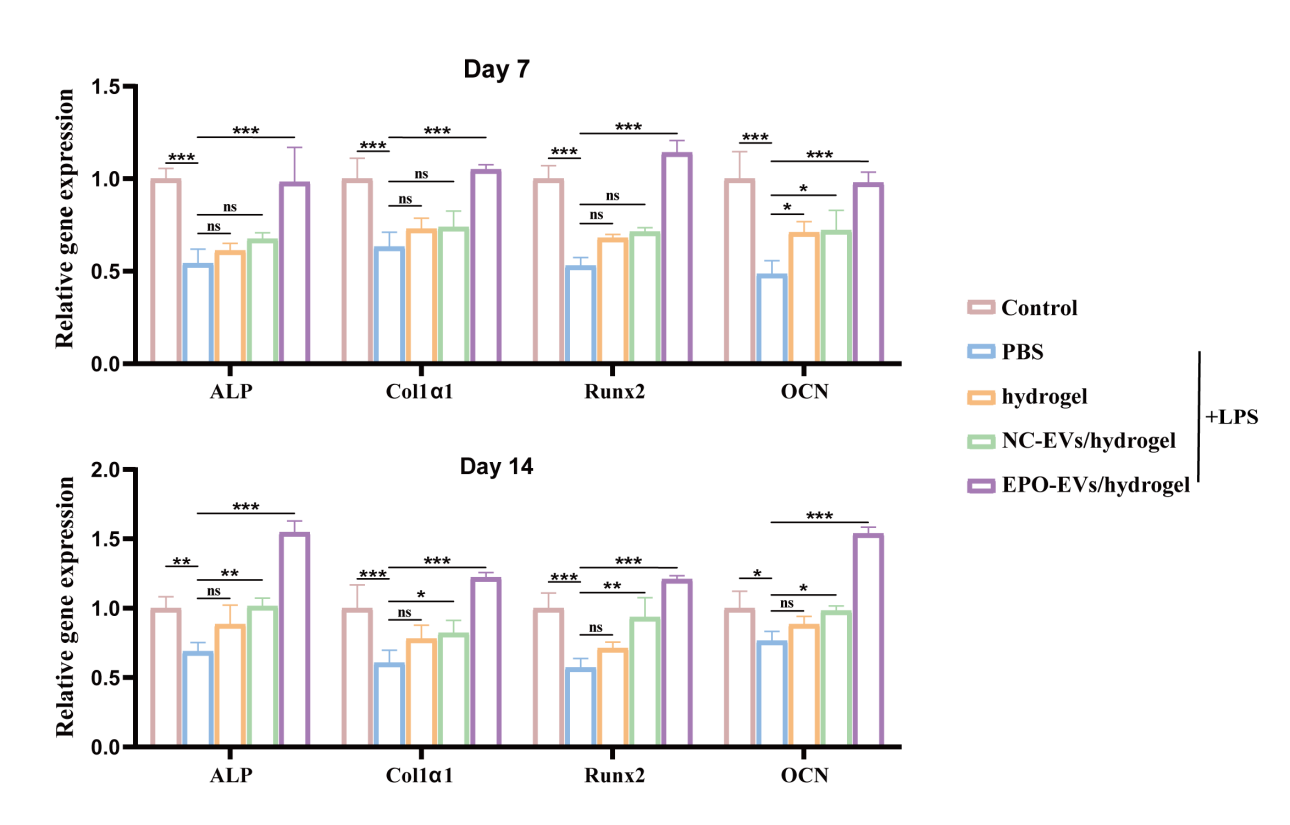
**

**Figure S3.** qPCR showed the expression of osteogenic-related genes (ALP, Col1α1, Runx2, and OCN) in mBMSCs from each group on day 7 and 14 (n = 3) (* p < 0.05, ** p < 0.01, *** p < 0.001). The data are analyzed using ANOVA and presented as mean value ± SD.


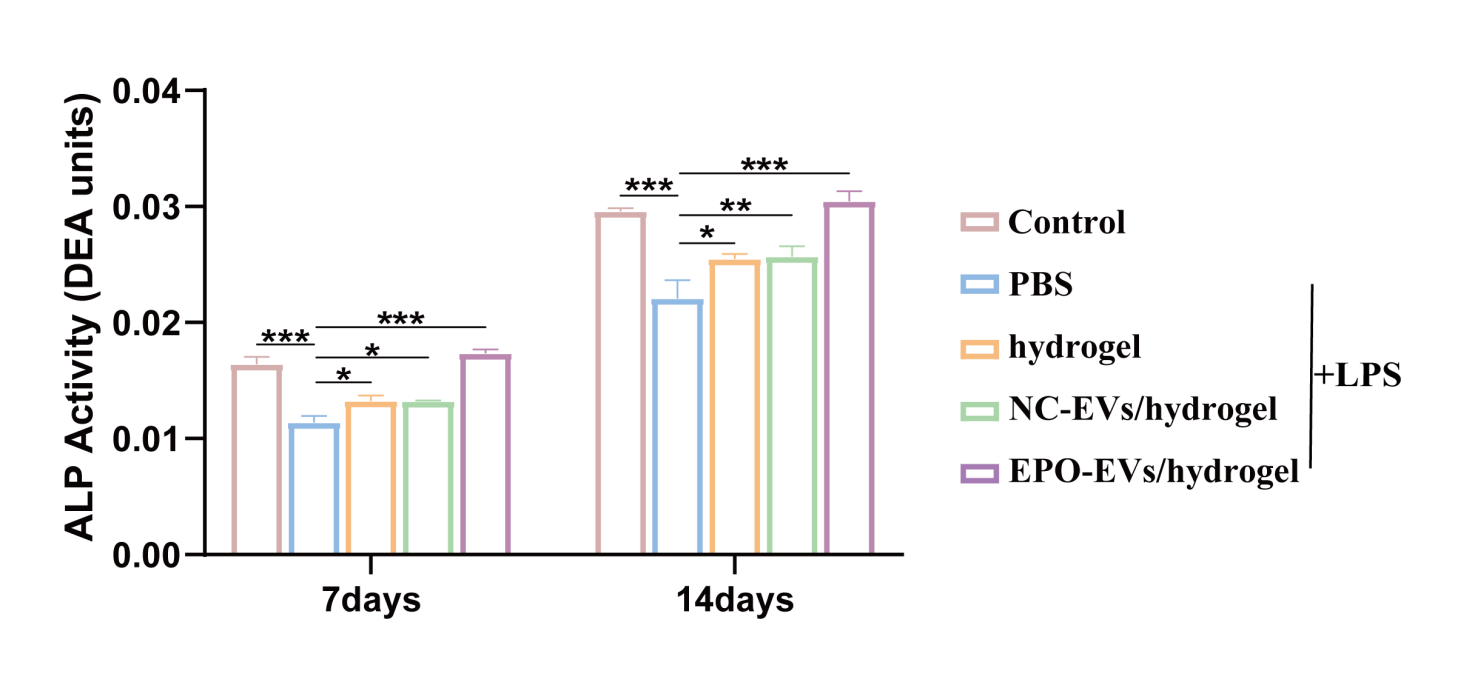
**Figure S4.** Quantitative analysis of ALP activity of mBMSCs on day 7 and 14 (n = 3) (* p < 0.05, ** p < 0.01, *** p < 0.001). The data are analyzed using ANOVA and presented as mean value ± SD.


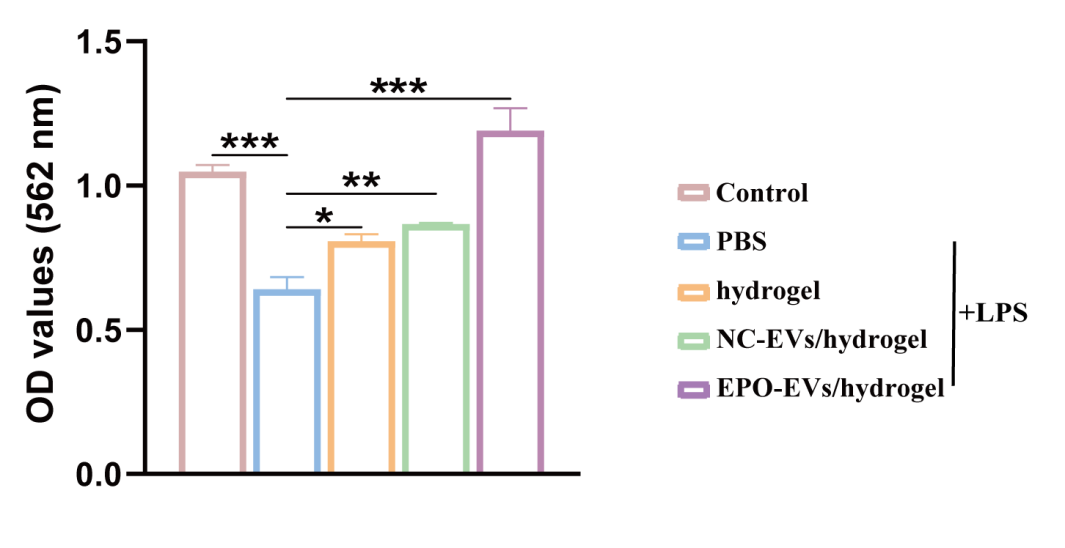


**Figure S5.** Quantitative analysis of ARS staining of mBMSCs on day 21 (n = 3) (* p < 0.05, ** p < 0.01, *** p < 0.001). The data are analyzed using ANOVA and presented as mean value ± SD.

**
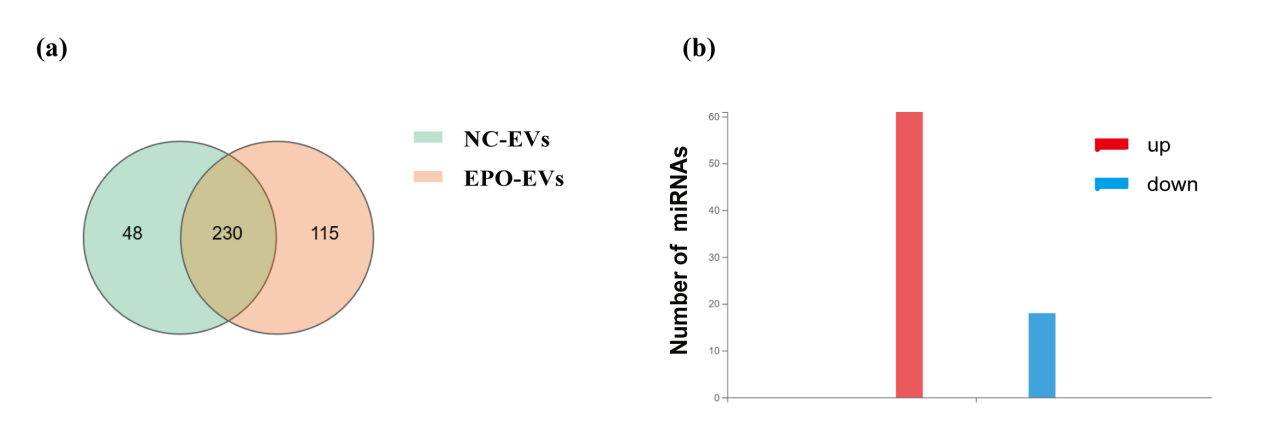
**

**Figure S6.** miRNA-sequencing analysis. (a) Overlapping miRNAs in the NC-EVs and EPO-EVs. (b) Differentially expressed miRNAs (DEMs) (|log_2_(fold change)| ≥ 2 and Q value < 0.05) between NC-EVs and EPO-EVs.

**
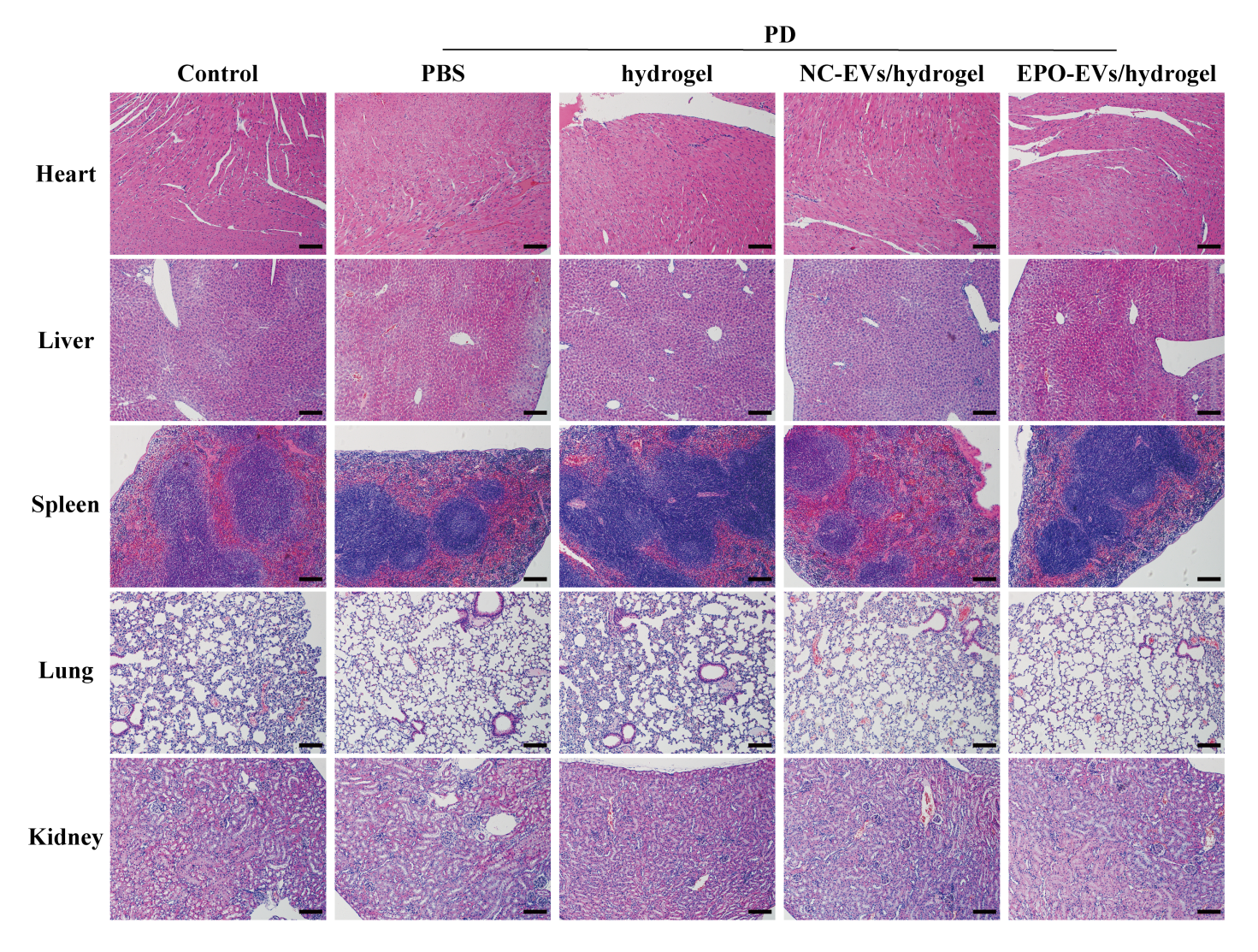
Figure S7.** Histological H&E staining of the major organs in each group. Scale bar = 100 μm.

**Supplementary Table 1. Information of antibodies.**

| Antibody Name | Company | Cat No |
| --- | --- | --- |
| HSP70 | Proteintech | 10995-1-AP |
| CD63 | Abcam | ab134045 |
| Runx2 | Abcam | ab236639 |
| OCN | Beyotime | AF6297 |
| EGFR | Beyotime | AF1330 |
| RhoA | Abcam | ab187027 |
| β-actin | proteintech | 10554-1-AP |
| HRP-conjugated Goat Anti-Rabbit IgG(H+L) | proteintech | SA00001-2 |
| HRP-conjugated Goat Anti-Mouse IgG(H+L) | proteintech | SA00001-1 |
| Cy3-labeled goat anti-rabbit IgG | Beyotime | A0516 |
| Alexa Fluor 555-phalloidin | Beyotime | C2203S |

**Supplementary Table 2. Osteogenesis-related primer sequences in this stud**y

| Gene | Primer sequence (5’ to 3′) |
| --- | --- |
| ALP | Forward: TGCCTACTTGTGTGGCGTGAA |
|  | Reverse: TCACCCGAGTGGTAGTCACAATG |
| Runx2 | Forward: TCAGCGTCAACACCATCATTC |
|  | Reverse: CCAGACCAGCAGCACTCCATA |
| Col1α1 | Forward: AGAACAGCGTGGCCT |
|  | Reverse: TCCGGTGTGACTCGT |
| OCN | Forward: GCTTGTGACGAGCTATCAGACCAG |
|  | Reverse: AGCTGCTGTGACATCCATACTTGC |
| GAPDH | Forward: TGTGTCCGTCGTGGATCTG |
|  | Reverse: TTGCTGTTGAAGTCGCAGGA |

**Supplementary Table 3. List of mmu-miRNA primer sequences**

| mmu-miRNA | Primer sequence (5’ to 3′) |
| --- | --- |
| mmu-miR-702-5p | GUGAGUGGGGUGGUUGGCAUG |
| mmu-miR-5107-5p | UGGGCAGAGGAGGCAGGGACA |
| mmu-miR-193a-5p | UGGGUCUUUGCGGGCAAGAUGA |
| mmu-miR-142a-5p | CAUAAAGUAGAAAGCACUACU |
| mmu-miR-449a-5p | UGGCAGUGUAUUGUUAGCUGGU |
| mmu-miR-1981-5p | GUAAAGGCUGGGCUUAGACGUGGC |
| mmu-miR-542-5p | CUCGGGGAUCAUCAUGUCACGA |
| mmu-miR-18a-3p | ACUGCCCUAAGUGCUCCUUCUG |
| mmu-miR-6913-5p | UGGGAGACCAGUCAGGUGUUGG |
| mmu-miR-196a-5p | UAGGUAGUUUCAUGUUGUUGGG |
| mmu-U6 | CGCTTCGGCAGCACATATAC |
